# Integrative analysis of metabolite and transcriptome reveals the biosynthetic pathway and candidate genes for iridoid glycoside biosynthesis in *Neopicrorhiza scrophulariiflora* (Pennell) D.Y.Hong

**DOI:** 10.3389/fpls.2025.1527477

**Published:** 2025-02-03

**Authors:** Ke Rao, Siyu Liu, Xiaohui Tang, Guofu Jia, Shaohua Yang, Chaoxiang Ren, Jin Pei

**Affiliations:** ^1^ State Key Laboratory of Southwestern Chinese Medicine Resources, Chengdu, Sichuan, China; ^2^ College of Pharmacy, Chengdu University of Traditional Chinese Medicine, Chengdu, Sichuan, China; ^3^ Sichuan Academy of Grassland Sciences, Chengdu, China; ^4^ Yunnan Provincial Academy of Agricultural Sciences of Alpine Economic Plant Research Institute, Yunnan, China

**Keywords:** *N. scrophulariiflora*, picroside-I, transcriptome, metabolites, biosynthetic pathway

## Abstract

*Neopicrorhiza scrophulariiflora* (Pennell) D.Y.Hong (*N. scrophulariiflora*) is an important wild medicinal plant that belongs to the Plantaginaceae family. Its main active ingredients, picroside I (P-I) and picroside II (P-II), possess anti-inflammatory, anticancer, and antibacterial properties. Due to overharvesting, *N. scrophulariiflora* resources are facing the risk of depletion, urgently requiring resource protection and rational utilization. However, the biosynthetic pathways and related genes of active compounds in *N. scrophulariiflora* have not been fully investigated. In this study, widely targeted metabolomics and RNA-seq technology were employed to perform a joint analysis of the metabolome and transcriptome in different tissues of *N. scrophulariiflora*, including the roots, stems, and leaves. A total of 196 flavonoids and 63 terpenoids were identified. Among the 158,254 annotated genes, 74 were annotated as related to iridoid synthesis. Using bioinformatics methods such as clustering analysis, phylogenetic tree construction, and weighted gene co-expression network analysis (WGCNA), 43 candidate genes were identified that may be involved in the biosynthesis of picroside-I and picroside-II, of which 26 genes were significantly correlated with the synthesis of picrosides and their intermediates. Transcriptome analysis revealed the expression patterns of differentially expressed genes, and metabolomic analysis revealed the distribution characteristics of metabolites in different tissues of *N. scrophulariiflora*. Through qRT-PCR validation, we found that three *NsF3H/NsF3D* genes, four *NsUGD/NsUPD* genes, one *Ns2HFD* gene, and three *NsSQM* genes may participate in the iridoid biosynthesis pathway. These findings provide important genetic and metabolomic information for an in-depth understanding of the biosynthetic mechanisms of iridoids and lay the foundation for the protection and sustainable utilization of *N. scrophulariiflora*.

## 1 Introduction

*Neopicrorhiza scrophulariiflora* (Pennell) D.Y.Hong (*N. scrophulariiflora*) is a perennial medicinal plant that belongs to the genus *Neopicrorhiza* of the family Plantaginaceae. It is distributed across southern Tibet, northwestern Yunnan, western Sichuan in China, and Nepal (Bantawa et al., 2010; Kafle et al., 2018). It is a congeneric plant with *Neopicrorhiza kurroa* Royle ex. Benth. (*N. kurroa*), and they are the only two species of this genus. Owing to overharvesting and the lack of organized cultivation in recent years, its habitat range has become increasingly narrow, resulting in a sharp decline in the population of *N. scrophulariiflora*. This species has been listed as an endangered species by the IUCN (Nayar and Sastri, 1990; Bantawa et al., 2011). Studies have shown that the secondary metabolites of *N. scrophulariiflora* possess various pharmacological effects, such as liver protection, ischemia–hypoxia protection, and anti-inflammatory, anti-asthmatic, and immunomodulatory effects (He and Lin, 2005a, He and Lin, 2005b; Cao, 2008; Li et al., 2010; Weng et al., 2016; Wang et al., 2017). The active components of *N. scrophulariiflora* include iridoids, phenylethanoids, and phenolic glycosides (Cao et al., 2012; He et al., 2012; Han et al., 2014). Among them, the iridoid glycosides picroside-I and picroside-II are monoterpene derivatives formed by the acylation of catalpol and phenolic acids. They are also unique active ingredients in this genus. According to the Chinese Pharmacopoeia (2020 edition), the total contents of picroside-I (P-I) and picroside-II (P-II) are medicinal indicators of *N. scrophulariiflora*.

P-I and P-II are iridoid glycosides of *N. scrophulariiflora*, and the synthesis of iridoid glycosides can be divided into two stages. In the first stage, geranyl diphosphate (GPP) is produced via the MVA (mevalonate) and MEP (methylerythritol phosphate) pathways. The key enzymes involved include 1-deoxy-D-xylulose-5-phosphate synthase (DXPS), 1-deoxy-D-xylulose-5-phosphate reductoisomerase (DXPR), acetoacetyl-CoA thiolase (ACTH), mevalonate kinase (MVK), and 4-(cytidine-5′-diphospho)-2-C-methylerythritol kinase (ISPE). In these two pathways, isopentenyl pyrophosphate (IPP) undergoes a series of reactions to convert into geranyl diphosphate (GPP) (Hampel et al., 2006; Tohge and Fernie, 2017). The second stage involves the iridoid biosynthesis pathway. At this stage, geranyl diphosphate (GPP) undergoes the removal of two phosphate groups to produce geraniol. Subsequently, geraniol is sequentially converted through a series of enzyme-catalyzed reactions to form 10-hydroxygeraniol, 10-oxogeranial, and iridodial, which ultimately participate in the synthesis of iridoid glycosides (Guo et al., 2024).

However, the downstream biosynthetic pathways of P-I and P-II have not been elucidated, and researchers speculated about the biosynthetic pathway of picrosides in *N. kurroa* (Shitiz et al., 2015). This pathway begins with the iridoid skeleton. The products generated from the iridodial and epi-iridodial undergo cyclization to form iridorials. The subsequent steps include the formation of an aldehyde group at the C-4 position, glucosylation at the C-1 position, and simultaneous oxidation of the aldehyde group at the C-4 position to form a carboxyl group, resulting in 7-deoxyloganic acid. This enzyme-catalyzed step is likely catalyzed by aldehyde dehydrogenase (ALD). Subsequently, 7-deoxyloganic acid is converted to mussaenosidic acid in a similar reaction catalyzed by flavanone 3-dioxygenase/hydroxylase (F3D). Mussaenosidic acid undergoes dehydration catalyzed by 2-hydroxyisoflavanone dehydratase (2HFD) to produce deoxygeniposidic acid, which then undergoes hydrolysis at C-10 to form geniposidic acid. Geniposidic acid undergoes decarboxylation at C-4, catalyzed by uroporphyrinogen decarboxylase (UPD/UGD), to produce bartsioside. Subsequently, hydroxylation occurs at C-6 to form aucubin, and epoxidation with an alcohol at C-10 catalyzed by squalene monooxygenase (SQM) produces catalpol. Finally, anthocyanin acyltransferase (ACT) catalyzes the conversion of catalpol to P-I and P-II (Kharb et al., 2022).

Studies have shown that the picroside content in different tissues of *N. kurroa* varies. The P-I content in leaf tissues was significantly higher than that in the stems and roots, whereas the P-II content in root tissues was higher than that in leaves. P-I and P-II were present in comparable amounts in the stems (Singh et al., 2011; Kumar et al., 2012). Given that most of the chemical components of *N. scrophulariiflora* are similar to those of *N. kurroa*, the study in *N. kurroa* provided a reference for screening candidate genes involved in the biosynthesis of iridoid compounds in *N. scrophulariiflora*. Owing to differences in taxonomic classification and unique growing environments, *N. scrophulariiflora* and *N. kurroa* likely exhibit distinct metabolite compositions and differentially expressed genes. To comprehensively understand the biological characteristics of plants in the genus *Neopicrorhiza*, conducting transcriptomic and metabolomic analyses is an effective research approach.

In this experiment, three different tissues of *N. scrophulariiflora*, namely, roots, stems, and leaves, were selected as materials. Widely targeted metabolomics and RNA-seq techniques were employed to analyze metabolite profiles in different tissues of *N. scrophulariiflora* and screen key genes involved in the biosynthesis of iridoid glycosides. This study revealed the potential molecular mechanisms of the iridoid biosynthesis pathway in *N. scrophulariiflora* and provided important genetic and metabolic information for further investigation in this plant. These findings offer new insights for the conservation and rational utilization of *N. scrophulariiflora* as a medicinal resource.

## 2 Materials and methods

### 2.1 Plant materials

Three different parts of *N. scrophulariiflora* (roots, stems, and leaves) were collected under consistent growth conditions with the same growth status. The collected samples were quickly frozen in liquid nitrogen and stored at −80°C. Each transcriptome and metabolome sample was prepared in triplicate.

### 2.2 Liquid chromatography analysis of *N. scrophulariiflora* compounds

The freeze-dried samples were crushed using a mixer mill (MM 400, Retsch) with a zirconia bead for 1.5 min at 30 Hz. 100 mg of lyophilized powder was dissolved with 1.2 mL of 70% methanol solution, vortexed 30 s every 30 min for six times in total, and the sample was placed in a refrigerator at 4°C overnight. After centrifugation (rotation speed 12,000 rpm, 3 min), the supernatant was aspirated, and the samples were filtered and analyzed using UPLC-MS/MS (UPLC, ExionLC™ AD). The analytical conditions were as follows: UPLC column, Agilent SB-C18 (1.8 µm, 2.1 mm × 100 mm). The mobile phase consisted of solvent A, pure water with 0.1% formic acid, and solvent B, acetonitrile with 0.1% formic acid. Sample measurements were performed using a gradient program that employed starting conditions of 95% A and 5% B. Within 9 min, a linear gradient of 5% A and 95% B was programmed, and a composition of 5% A and 95% B was maintained for 1 min. Subsequently, the composition was adjusted to 95% A and 5.0% B within 1.1 min and kept for 2.9 min. The effluent was alternately connected to an ESI-triple quadrupole linear ion trap (QTRAP)-MS.

### 2.3 Qualitative and quantitative metabolite analyses

Metabolite data were log2-transformed for statistical analysis. Metabolites from nine samples were used for hierarchical clustering analysis (HCA), principal component analysis (PCA), and orthogonal partial least squares discriminant analysis (OPLS-DA) using R software (www.r-project.org) to study metabolite accession-specific accumulation. The *p* and fold change values were set to 0.05 and 2.0, respectively. Venn diagrams were used to determine the number of different metabolites present.

### 2.4 RNA extraction and RNA-Seq

Total RNA was extracted from the samples using an RNAprep Pure Plant kit (DP441; Tiangen, China). RNA quality was detected using a NanoPhotometer spectrophotometer (IMPLEN, CA, USA), Qubit 2.0 Fluorometer (Life Technologies, CA, USA), and Agilent Bioanalyzer 2100 system (Agilent Technologies, CA, USA). Poly (A) mRNA was enriched using magnetic beads containing oligo (dT). The mRNA was randomly fragmented. First-strand cDNA was synthesized using the M-MuLV reverse transcriptase system. The RNA strand was then degraded by RNase H, and the second-strand cDNA was synthesized using DNA polymerase. Double-stranded cDNAs were ligated to the sequencing adapters. The cDNAs (~200 bp) were screened using AMPure XP beads. After amplification and purification, cDNA libraries were obtained and sequenced using an Illumina NovaSeq 6000 system.

Raw reads were transformed from raw image data by CASAVA-based recognition. To obtain high-quality data, adapters of sequences were cut, and low-quality reads with ≥5 uncertain bases or with over 50% Qphred ≤20 bases were removed using fastp. The GC content of the clean reads was calculated. The Q20 and Q30 values were also produced by FastQC to evaluate the base quality. Gene expression levels were determined using the fragments per kilobase per million reads (FPKM) method (Chen et al., 2018).

### 2.5 Weighted gene co-expression network analysis

WGCNA was conducted with normalized read counts of the 31,427 differentially expressed genes (DEGs) using the R package (WGCNA 1.71, Version-3.5.1) (Zhang and Horvath, 2005; Langfelder and Horvath, 2008) with the following parameters: three biological replicates of three different tissues were used to ensure that the scale-free topology index was >0.8. Topological overlap matrix (TOM)-signed, power *β* = 14, minimal module size = 30, reassign threshold = 0, and branch merge cut height = 0. The eigengene (the first principal component of a given module) value was calculated and used for association analysis between a module and the measured metabolites from the three different tissues.

A hierarchical clustering heatmap was generated by converting normalized expression values into Z-scores using the formula Z = (Log2 (normalized count+1)−mean)/standard deviation. For metabolites, Z-scores were calculated using the same formula; however, the amount of metabolite was used instead of the normalized count.

### 2.6 Real-time PCR analysis

Purified RNA (1 µg for each sample) was reverse-transcribed to first-strand cDNA using a cDNA Reverse Transcription Kit (PrimeScript™ RT Master Mix, Takara) according to the manufacturer’s instructions. The primers used to amplify the screened genes, *NsSQM1*, *NsSQM4*, *NsSQM5*, *NsUGD5*, *NsUGD7*, *NsUPD2*, *NsUPD3*, *Ns2HFD4*, *NsF3D3*, *NsF3H5*, and *NsF3H6*, were designed by Primer Premier 5 software. 26SrRNA were used as internal reference genes (**Supplementary Table S1**). qRT-PCR was conducted using a ChamQ SYBR qPCR Master Mix kit (Vazyme) and a C1000 Touch™ Thermal Cycler system (Bio-Rad). Relative transcript levels were calculated according to the 2^−ΔΔCt^ method (Livak and Schmittgen, 2001; Mortazavi et al., 2008). Three biological and technical replicates were used for each experiment.

## 3 Results

### 3.1 Differences in metabolite between different tissues of *N. scrophulariiflora*

**Figure 1:**
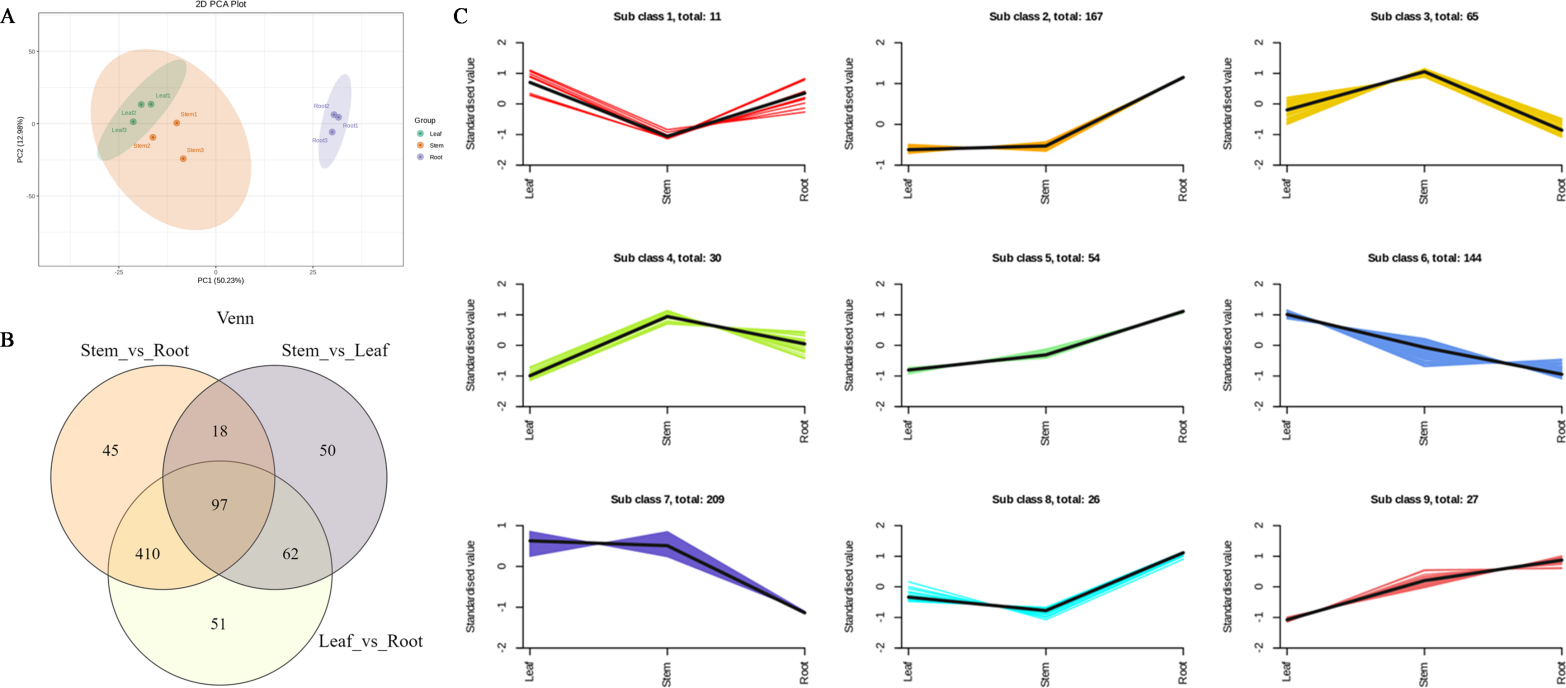
Differential metabolites analysis. **(A)** Principal component analysis (PCA) of LC-MS/MS results from different tissue samples. **(B)** Statistics for the DAMs for the different comparisons (R vs. S, S vs. L, and L vs. R) in *N. scrophulariiflora* tissues. **(C)** Line chart plot of the K-means clustering analysis of the differential metabolites.

The total ion current (TIC) of the mixed quality control (QC) samples showed the same retention time and peak area (**Supplementary Figure S1**). Correlation analysis between samples showed good biological repetition, and all QC samples had good reproducibility (**Supplementary Figure S2**). In this study, nine samples were selected and divided into three groups for metabolomic analysis. A total of 1,085 metabolites were detected and divided into seven groups, namely, phenolic acids, flavonoids, quinones, lignans, alkaloids, terpenes, and other compounds (**Supplementary Figure S3**). Among them, 196 flavonoids and 63 terpenes were identified (**Supplementary Table S2**). Principal component analysis (PCA) results showed that root tissues had a different trend of metabolome separation with leaf and stem tissues. Notably, root tissues exhibit a broader divergence in metabolite profiles compared with leaf and stem tissues (**Figure 1A**). Hierarchical cluster analysis (HCA) was performed on the accumulation patterns of metabolites in different samples (**Supplementary Figure S4**).

In each comparison group, differentially accumulated metabolites (DAMs) with variable importance in projection (VIP) ≥1 and fold change (FC) ≥2 or ≤0.5 were selected. DAMs in different tissues of *N. scrophulariiflora* were analyzed. A total of 733 DAMs were obtained from the comparison groups (**Supplementary Table S3**). The number of DAMs in each group is shown in **Figure 1B**, and the DAMs in each comparison group are summarized in **Supplementary Table S4.** 620, 570, and 227 DAMs were identified in the root vs. leaf, root vs. stem, and leaf vs. stem comparisons, respectively. In the root vs. leaf comparison, 257 DAMs were upregulated and 363 were downregulated. In the root vs. stem comparison, 215 DAMs were upregulated and 355 were downregulated. In the leaf vs. stem comparison, 66 DAMs were upregulated and 161 were downregulated. These results indicate that the differences in DAMs between root and leaf, as well as root and stem, are relatively large, whereas the differences between leaf and stem are relatively smaller. To investigate the relative changes in DAM abundance across different groups, a K-means clustering analysis was performed. This analysis grouped the DAMs into nine clusters (**Figure 1C**). Among these, 364 metabolites were found to be significantly increased in leaves, the majority of which were phenolic acids and flavonoids, such as gentisic acid, 6-hydroxykaempferol-7-*O*-glucoside, luteolin-7-*O*-(6"-malonyl) glucoside, 6"-*O*-malonylgenistin, quercetin-7-*O*-(6"-malonyl)glucoside, and tricin-7-*O*-(6"-*O*-malonyl) glucoside. In stems, a total of 304 metabolites were significantly increased, most of which were also phenolic acids, such as 4-hydroxybenzoic acid, 6-*O*-acetylarbutin, 6-[4-(acetyloxymethyl)phenoxy]-4-hydroxyoxane-2-carboxylic acid, gentisic acid 2-*O*-(6"-*O*-feruloyl)glucoside, and 1′-*O*-(3,4-dihydroxyphenethyl)-*O*-caffeoyl-glucoside. In roots, 274 metabolites were significantly increased, the majority of which were flavonoids and phenolic acids. Notably, 410 metabolites showed significant differences in both the root vs. leaf and root vs. stem comparisons.

Further research revealed that there are differences in the content of terpenoid metabolites in *N. scrophulariiflora*. A total of 63 terpenoid compounds were detected, including 20 iridoid glycosides, 8 monoterpenes, 3 diterpenes, and 29 triterpenes. In the comparison between roots and leaves, 46 terpenoid compounds showed significant differences, with 22 terpenoid compounds upregulated and 24 terpenoid compounds downregulated in the roots (**Supplementary Table S5**).

The content of P-I, P-II, and their presumed precursor substances was measured, revealing that precursor products (such as 7-deoxyloganic acid, vanillic acid, cinnamic acid, and protocatechuic acid) and P-I were significantly higher in leaves and stems than in roots, whereas ferulic acid, 3,4-dimethoxy cinnamic acid, and P-II were higher in roots than in leaves and stems. For example, the P-I content decreased 33-fold in the roots, whereas that of P-II increased 8-fold in the roots. In addition, the precursor compound for picroside synthesis, 7-deoxyloganic acid, decreased 60-fold in the roots.

Moreover, the accumulation of the following 10 iridoid glycosides was higher in leaf tissues compared with other samples: rehmannioside J, 8-epiloganic acid, geniposidic acid, acteoside, 8-*O*-feruloylharpagide, *β*-ionone, 7-deoxyloganic acid, 6-*O*-(4-methoxybenzoyl) catalpol, and rehmannioside A. Conversely, the following five iridoid glycosides accumulated more in root tissues than in the others: amphicoside, cistanoside A, picroside-III (6-*O*-feruloylcatalpol), veronicoside, and rehmannioside B.

### 3.2 Transcriptome analysis of *N. scrophulariiflora*

**Figure 2:**
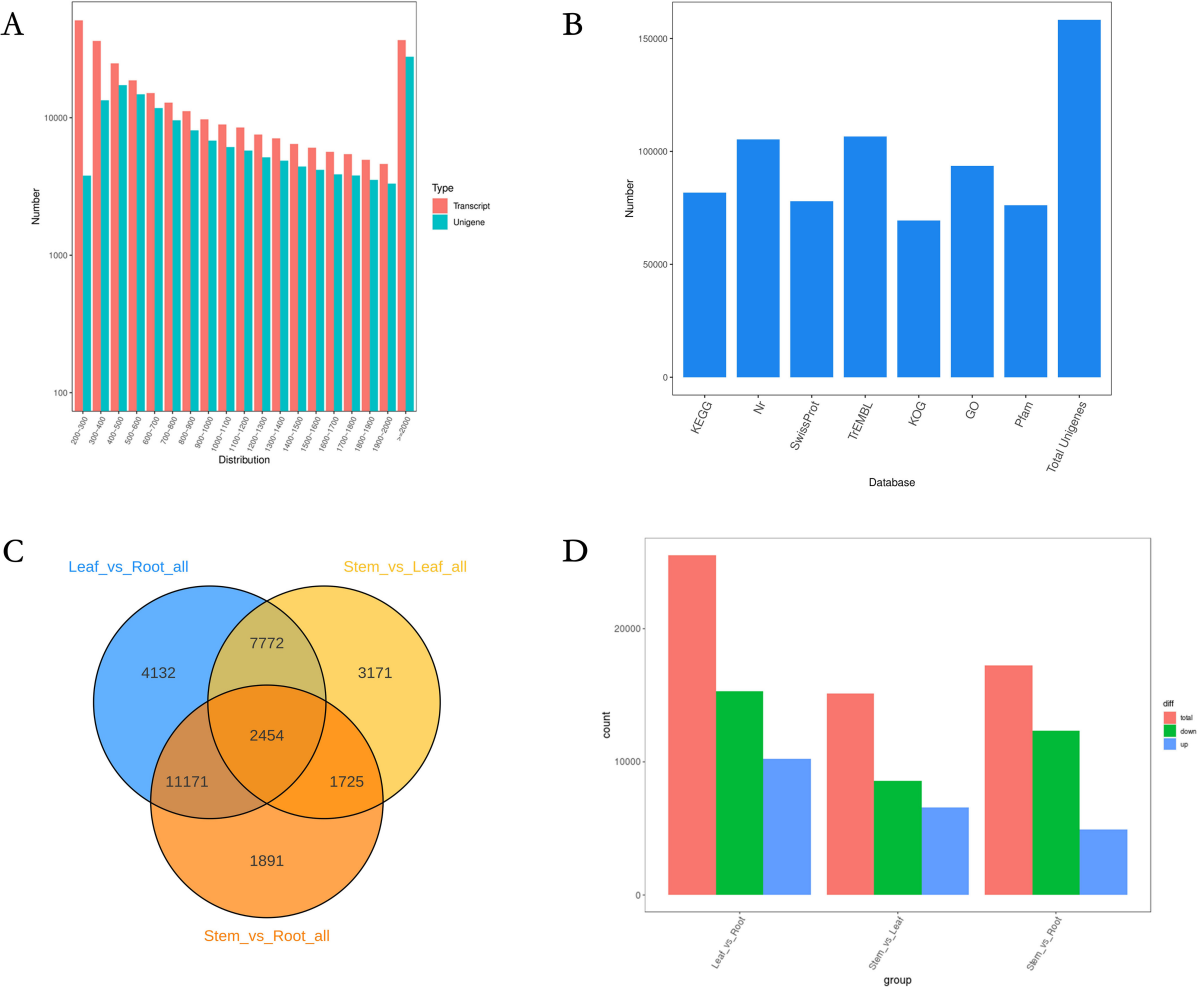
Construction of the full-length transcriptome of *N. scrophulariiflora* tissues. **(A)** Length distribution of genes. **(B)** Annotation of genes to the KEGG, NR, Swiss-Prot, GO, COG/KOG, and TrEMBL databases. **(C)** Venn diagram of the DEGs for the different comparisons. **(D)** Number of up- and downregulated DEGs in the different comparisons.

RNA-seq was used to obtain the transcriptomes of three different tissue samples. Raw data were submitted to NCBI (BioProject ID: PRJNA1171481). After DynamicTrim and LengthSort filtering, the average length and ratio of trimmed to raw reads were 1,010 bp and 97.85%, respectively. A total of 99.16 Gb of clean data was obtained. The Q30 fraction of each sample was greater than or equal to 88.41%. Among transcripts, the most common length range was 300 nt–2,000 nt, with an average length of 1,623 bp (**Figure 2A**). Sequences encoding peptide chains that were too short (less than 50 amino acid residues) or contained only a single exon were filtered out. Moreover, 158,284 genes were mined (**Supplementary Table S6**).

In this experiment, DIAMOND (Buchfink et al., 2015) BLASTX software was used to annotate the UniGene sequences with KEGG, NR, Swiss-Prot, GO, COG/KOG, and TrEMBL databases, and finally, 158,284 genes were annotated (**Figure 2B**). Among the 158,284 genes, 113,602 were annotated with at least one hit in the KEGG, NR, SwissProt, GO, COG/KOG, or TrEMBL databases (**Supplementary Table S7**). Considering the differences in iridoid glycoside content among the three tissue samples, this study investigated three comparison groups, leaf versus root tissue, stem versus leaf tissue, and stem versus root tissue, to screen for DEGs (**Figures 2C, D**). In comparisons between leaf and root tissues, stem and leaf tissues, and stem and root tissues, 25,529 DEGs (10,215 upregulated and 15,314 downregulated), 15,122 DEGs (6,554 upregulated and 8,568 downregulated), and 17,241 DEGs (4,925 upregulated and 12,316 downregulated) were identified, respectively.

### 3.3 Analysis of differentially expressed genes among different tissues of *N. scrophulariiflora*

**Figure 3:**
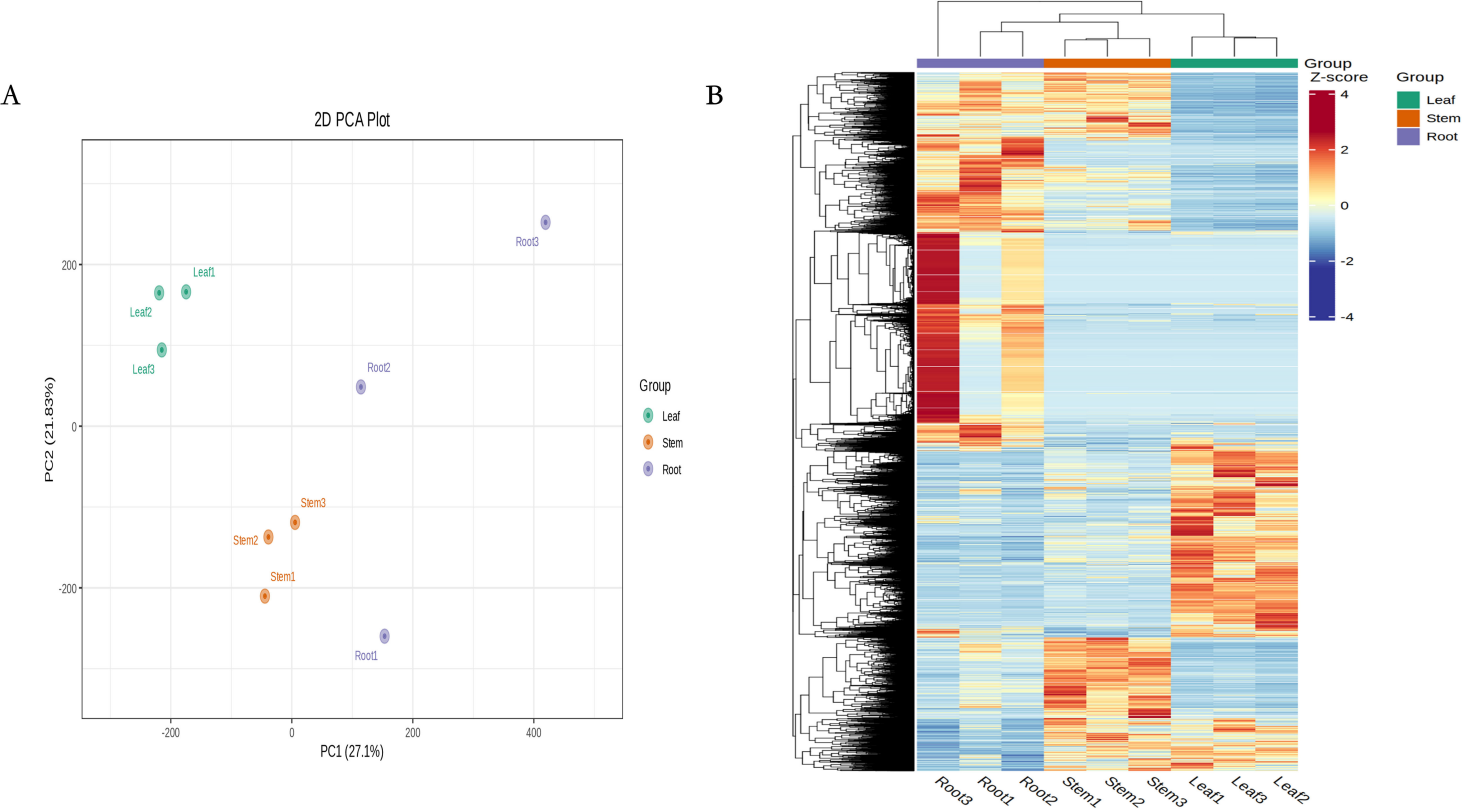
RNA-seq data expression profiles of *N. scrophulariiflora* tissues. **(A)** PCA of transcriptome data from the samples of three *N. scrophulariiflora* tissues. **(B)** Heatmap based on the hierarchical clustering analysis.

According to PCA and clustering heat maps, there were significant differences between the different tissues in the transcriptome (**Figure 3A, B**). According to the annotation results of the Nr database, most genes in the *N. scrophulariiflora* transcriptome had the highest homology with *Paulownia fortunei*, with 33,645 genes accounting for 31.95%, followed by *Buddleja alternifolia*, with 8,036 genes accounting for 7.63%. The species with higher homology were all annotated to the *Scrophulariaceae* family (**Supplementary Figure S5**). To study the functional relationship between DEGs and their related biological processes, the DEGs were mapped to GO terms. The enriched differentially expressed genes obtained from the analysis consisted of biological process (BP), cellular component (CC), and molecular function (MF). In the GO analysis of the “biological process” category, the most enriched terms in the upregulated and downregulated DEGs of the three groups were “cellular process” and “metabolic process” (**Supplementary Figure S6**). According to the KOG database, 106,982 genes were divided into 25 functional categories, with the largest and smallest categories being “general function prediction only” (14,075 genes) and “cell motility” (38 genes), respectively (**Supplementary Figure S7**).

**Figure 4:**
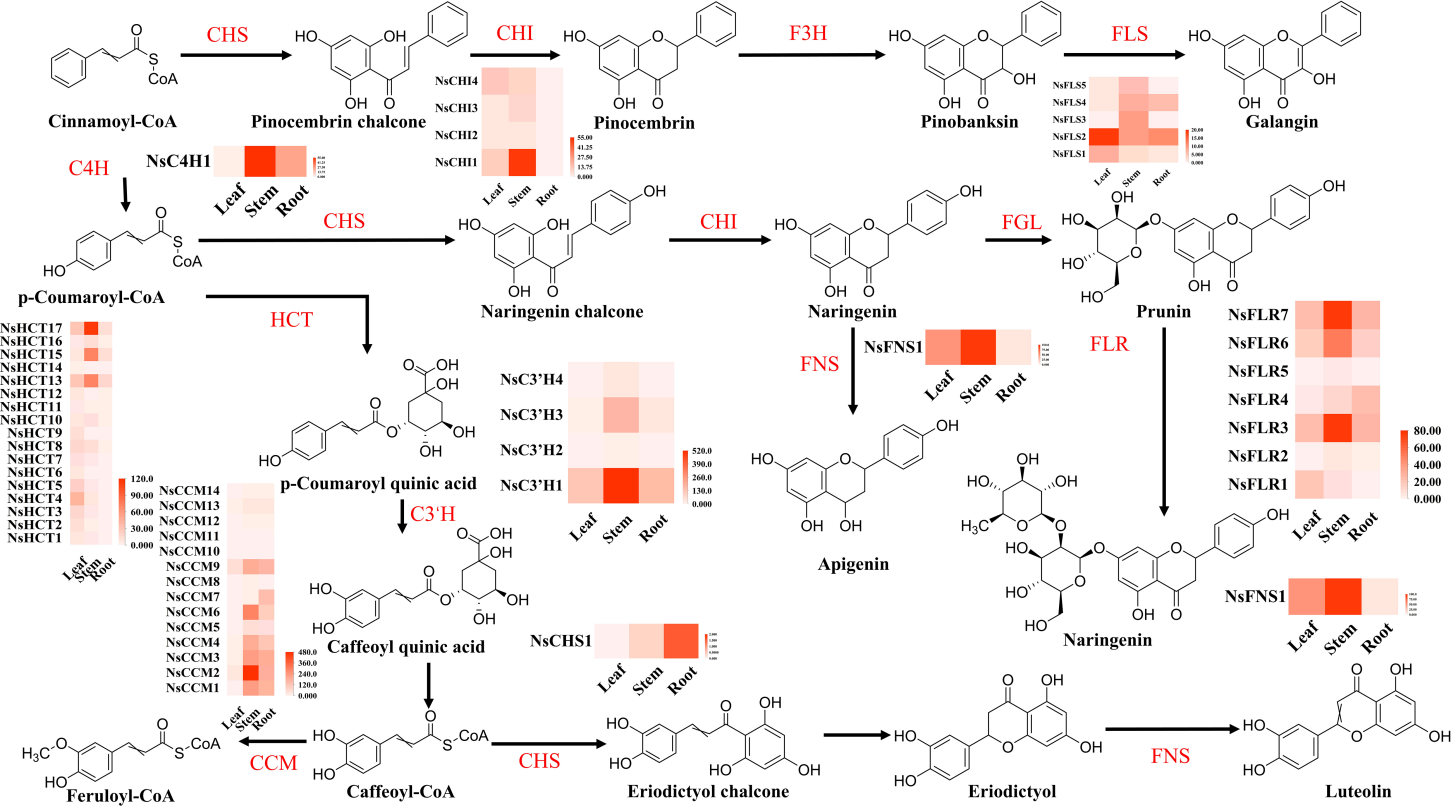
Flavonoid biosynthesis in different tissues of *N. scrophulariiflora*. Trans-cinnamate 4-monooxygenase (C4H), chalcone synthase (CHS), chalcone isomerase (CHI), flavonol synthase (FLS), shikimate *O*-hydroxycinnamoyltransferase (HCT), 5-*O*-(4-coumaroyl)-D-quinate 3′-monooxygenase (C3′H), caffeoyl-CoA *O*-methyltransferase (CCM), flavone synthase I (FNS), naringenin 3-dioxygenase (F3H), flavanone 7-*O*-beta-glucosyltransferase (FGL), and flavanone 7-*O*-glucoside 2"-*O*-beta-L-rhamnosyltransferase (FLR) were identified.

DEGs from the three groups were functionally classified using KEGG pathways. The results showed that the seven main classification pathways for the three groups of DEGs were as follows: “metabolism of terpenoids and polyketides,” “biosynthesis of secondary metabolites,” “phenylpropanoid biosynthesis,” “photosynthesis,” “pyruvate metabolism and dicarboxylate metabolism,” “carbon metabolism,” and “porphyrin metabolism” (**Supplementary Figure S8**). Most genes related to the phenylpropanoid and terpenoid biosynthesis pathways were significantly differentially expressed in all three comparisons. Among the top 20 most enriched pathways in the three comparison groups, “flavonoid biosynthesis,” “flavone and flavonol biosynthesis,” and “phenylpropanoid biosynthesis” showed significant enrichment. In the comparison between leaves and roots, most genes in the flavonoid metabolism pathways, especially flavonoid biosynthesis genes, showed significant changes in the roots (**Figure 4**). For example, *NsCHS1*, *NsFLS1*, *NsFLS2*, *NsFNS1*, *NsCCM1*, and *NsCCM3* were all significantly and differentially expressed in the roots. In the comparison between stems and roots, most flavonoid pathway genes were upregulated, such as *NsC3’H1*, *NsC3’H2*, *NsFLS3*, *NsFLS5*, *NsHCT13*, and *NsHCT17*; however, some flavonoid compound biosynthesis genes such as *NsCHS1* and *NsCHI1* were downregulated. Only *NsFLS3* and *NsFNS1* were downregulated in stems and leaves. In this pathway, metabolites (galangin, prunin, trans-5-*O*-(*p*-coumaroyl) shikimate, chlorogenic acid, and apigenin) accumulated significantly in the leaves and stems, which may be related to the high expression of genes in these tissues (**Supplementary Table S8**).

### 3.4 Identification of candidate genes involved in picroside biosynthesis

**Figure 5:**
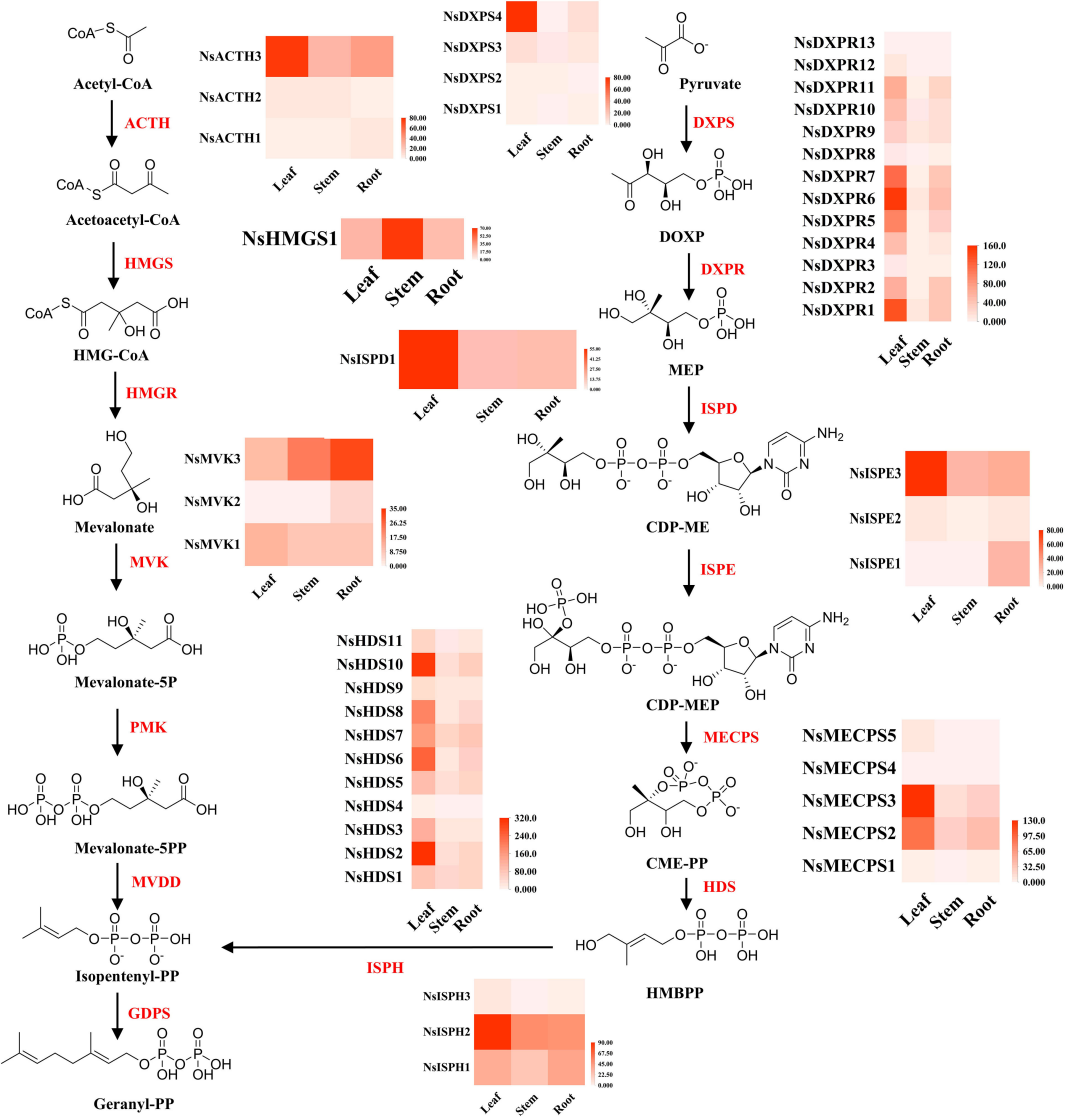
MVA and MEP biosynthesis in different tissues of *N. scrophulariiflora*. 1-Deoxy-D-xylulose-5-phosphate synthase (DXPS), 1-deoxy-D-xylulose-5-phosphate reductoisomerase (DXPR), 2-C-methylerythritol 4-phosphate cytidyl transferase (ISPD), 4-(cytidine-50-diphospho)-2-C-methylerythritol kinase (ISPE), 2-C-methylerythritol-2,4-cyclophosphate synthase (MECPS), 1-hydroxy-2-methyl-2-(E)-butenyl 4-diphosphate synthase (HDS), 1-hydroxy-2-methyl-2-(E)-butenyl 4-diphosphate reductase (ISPH), acetoacetyl-CoA thiolase (ACTH), hydroxymethyl glutaryl CoA synthase (HMGS), hydroxymethyl glutaryl CoA reductase (HMGR), and mevalonate kinase (MVK) were identified.

P-I and P-II are the most important secondary metabolites in *N. scrophulariiflora* and play decisive roles in its hepatoprotective functions. The upstream stages of the iridoid biosynthesis pathway include the MEP and MVA metabolic pathways. In the MEP pathway, *1-deoxy-D-xylulose-5-phosphate synthase* (DXPS), *1-deoxy-D-xylulose-5-phosphate reductoisomerase* (DXPR), *2-C-methylerythritol 4- phosphate cytidyl transferase* (ISPD), *4-(cytidine-50-diphospho)-2-C-methylerythritol kinase*, *2-C-methylerythritol-2,4- cyclophosphate synthase* (MECPS), *1-hydroxy-2-methyl-2-(E)-butenyl 4-diphosphate synthase* (HDS), and *1-hydroxy-2-methyl-2-(E)-butenyl 4-diphosphate reductase* (ISPH) were expressed at significantly higher levels in leaf tissues than in roots and stems. In the MVA pathway, *acetoacetyl-CoA thiolase* (ACTH), *hydroxymethyl glutaryl CoA synthase* (HMGS), *hydroxymethyl glutaryl CoA reductase* (HMGR), and *mevalonate kinase* (MVK) expression was significantly higher in the roots and stems than in the leaves (**Figure 5**).

**Figure 6:**
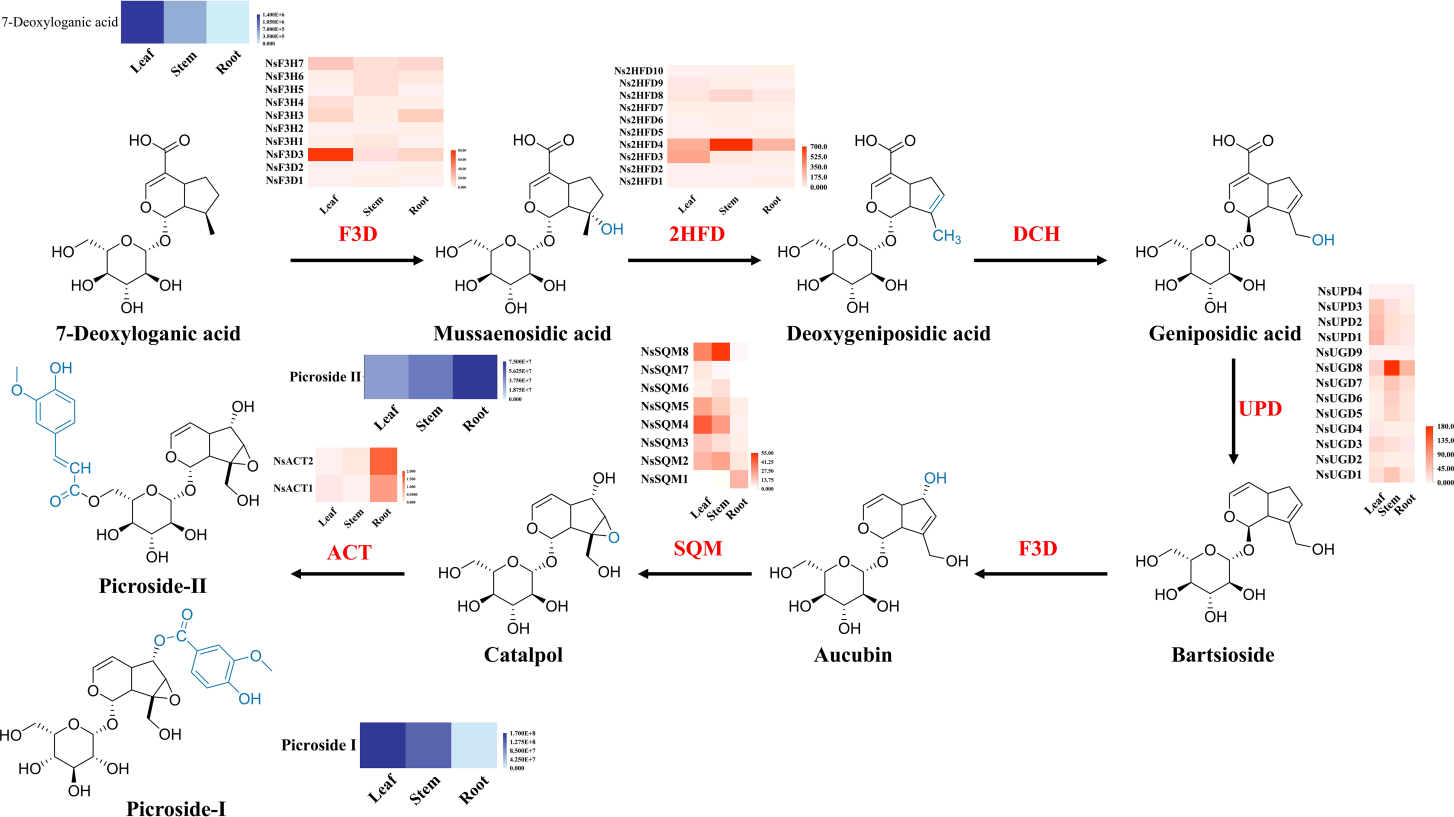
Biosynthesis of iridoid in *N. scrophulariiflora*. Heatmaps of the relative expression of genes encoding enzymes involved in iridoid synthesis in different tissues. 2-Hydroxyisoflavanone dehydratase (2HFD), uroporphyrinogen decarboxylase (UPD/UGD), flavanone 3-dioxygenase/hydroxylase (F3D), squalene monooxygenase (SQM), and anthocyanin acyltransferase (ACT) were identified.

Based on the functional annotation results, 43 genes related to the biosynthesis pathway of iridoid were identified. These 43 genes were categorized into five gene families, namely, 10 *2-hydroxyisoflavanone dehydratase* (2HFD) genes, 13 *UDP-glucose dehydrogenase* (UPD/UGD) genes, 10 *flavanone 3-hydroxylase/dioxygenase* (F3H) genes, 8 *squalene monooxygenase* (SQM) genes, and 2 *anthocyanin acyltransferase* (ACT) genes (**Figure 6**).

### 3.5 Integrative analysis of transcriptome and metabolome sequencing

**Figure 7:**
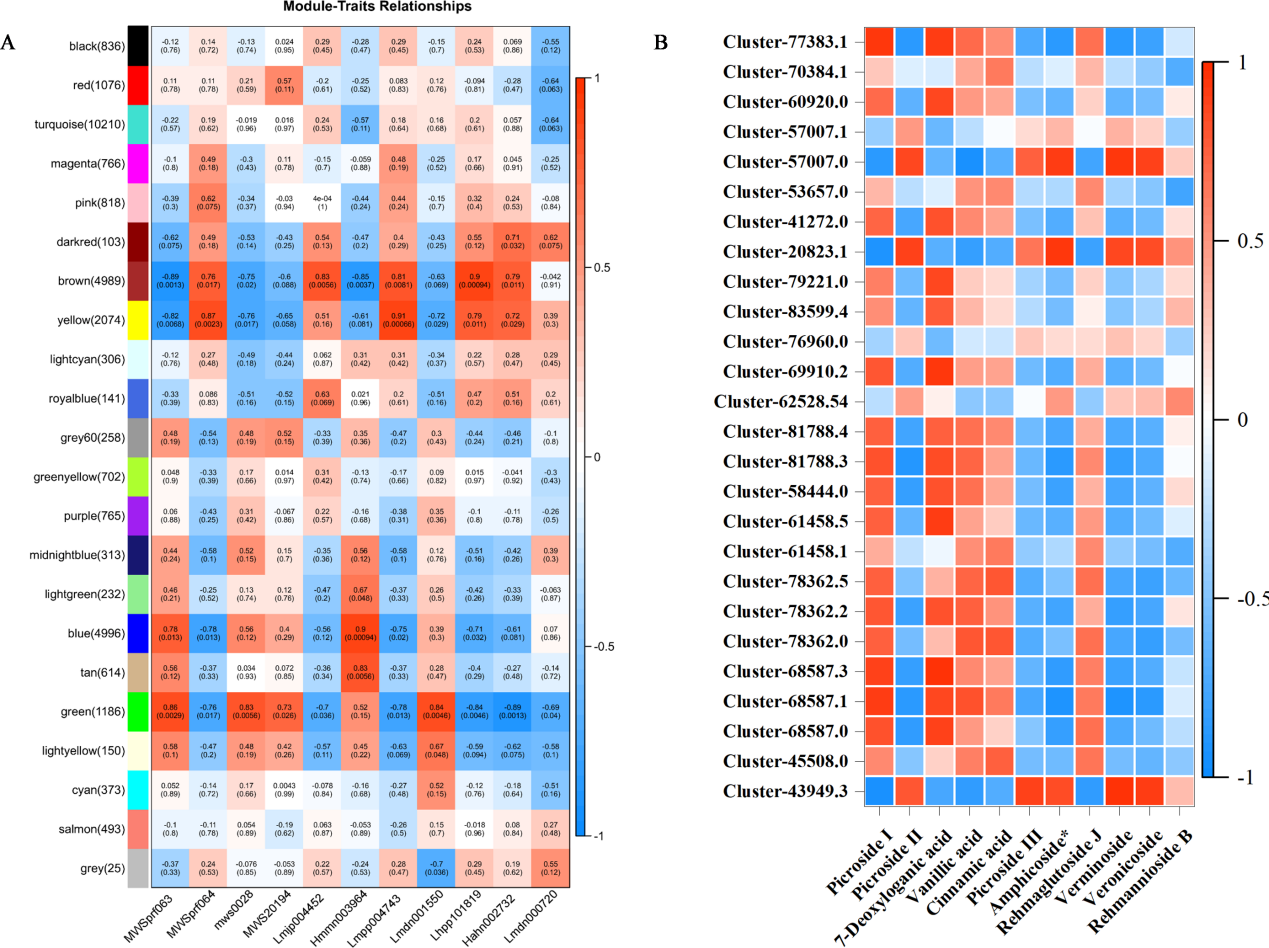
**(A)** WGCNA-based screening for hub genes related to iridoid biosynthesis. **(B)** Heatmaps showing an association between gene and 11 metabolites derived from weighted gene co-expression network analysis (WGCNA).

To further investigate the relationship between genes and metabolites in the leaf, root, and stem tissues, weighted gene co-expression network analysis (WGCNA) of the transcriptome and metabolome was performed to identify the co-expressed gene modules and key modules involved in iridoid biosynthesis. Based on the expression patterns, 22 co-expression modules were identified (**Figure 7A**). Among them, 26 genes related to the biosynthetic pathways of iridoids were screened. The analysis examined the correlation between the gene matrices of different modules and the iridoid compound content, with the correlation and corresponding *p*-values presented numerically in the grid where the modules intersected with the traits. Based on the “module–trait” correlation analysis, the green module was found to be significantly positively correlated with five iridoid glycoside components. This module contained 1,186 genes, three of which were annotated in the iridoid biosynthesis pathway. In addition, the brown module was significantly positively correlated with five other iridoid glycoside components. This module contained 4,989 genes, two of which were annotated to the iridoid biosynthesis pathway.

We further explored the correlation between 32,316 DEGs and 11 iridoid metabolites. The results showed that 26 genes were correlated with 11 metabolites in the iridoid pathway (**Figure 7B**). Among them, *NsSQM1*, *Ns2HFD1*, and *Ns2HFD5* were significantly correlated with P-II, and *NsUGD3*, *NsUPD2*, *NsUPD3*, *NsF3H4*, and *Ns2HFD9* were significantly correlated with P-I and 7-deoxyloganic acid.

### 3.6 Validation of gene expression profiles using qRT-PCR

**Figure 8:**
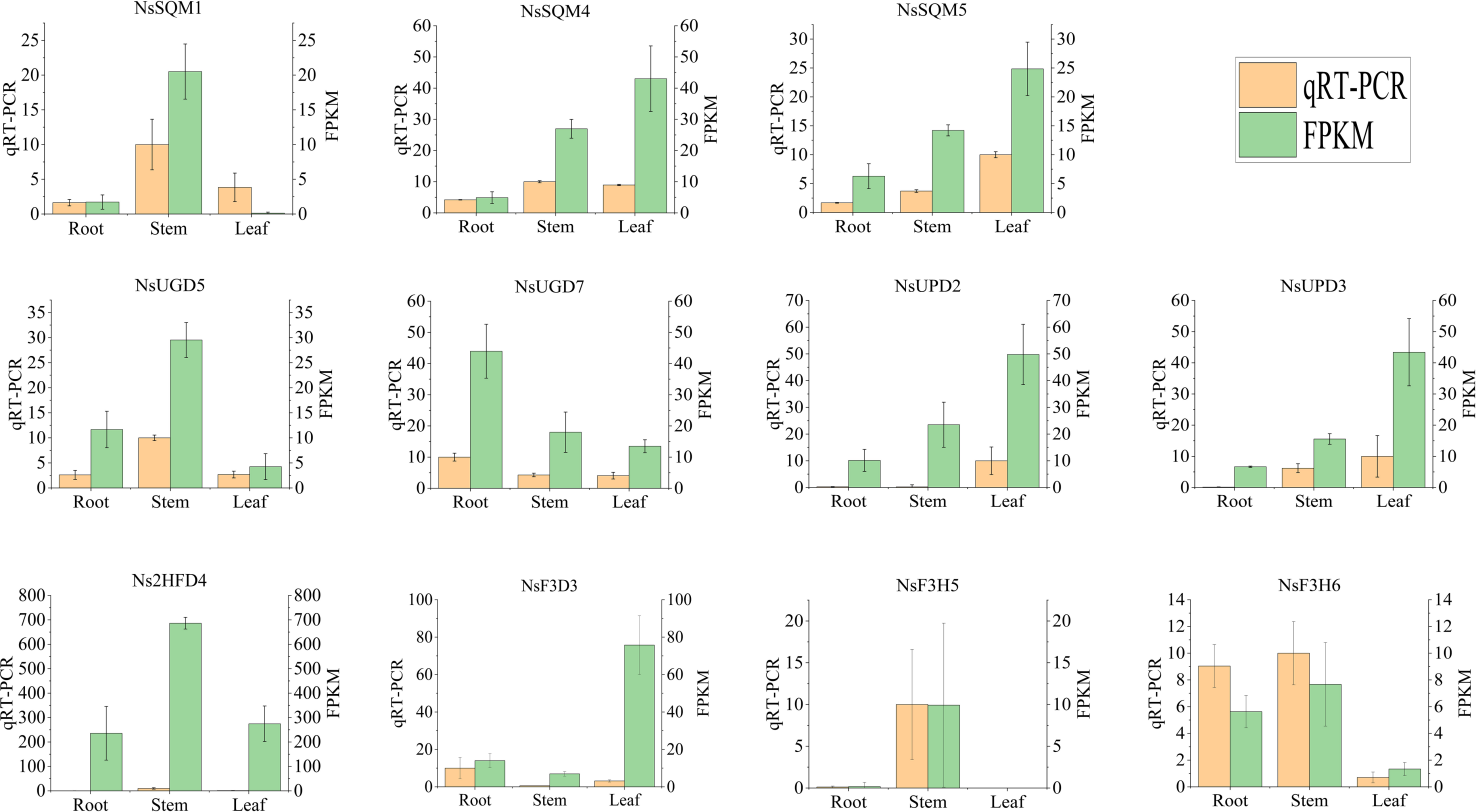
Expression of iridoid biosynthesis-related genes quantified by RNA-seq and qRT-PCR analyses. The y-axis represents the FPKM values of the genes from the RNA-seq data and the relative gene expression levels analyzed by qRT-PCR. The error bars indicate the standard error for three independent replicates.

To further validate the expression profiles of the genes from the Illumina sequencing analysis, 11 genes with high expression levels and large differences in expression were selected for qRT-PCR. qRT-PCR results showed that the relative expression of *NsUPD2*, *NsUPD3*, *NsSQM4*, and *NsSQM5* in the leaf tissue was significantly higher than that in the root tissue, and the relative expression of *NsF3H6* and *NsUGD7* in the root tissue was significantly higher than that in the leaf tissue. The expression patterns of the 11 genes were consistent with the sequencing data, indicating that the RNA-Seq data were reliable (**Figure 8**).

A phylogenetic analysis of four gene families was conducted, namely, *F3D/F3H*, *2HFD*, *UGD/UPD*, and *SQM*. For comparison, the results indicated that one gene, *Ns2HFD1*, was classified as a *2HFD* gene responsible for converting mussaenosidic acid to deoxygeniposidic acid. *Ns2HFD1* is more closely related to the *2HFD* gene in *Glycine max* (**Supplementary Figure S10**). The other three genes *NsF3D3*, *NsF3H5*, and *NsF3H6*, were classified as *F3H/F3D* genes responsible for converting bartsioside to aucubin or converting 7-deoxyloganic acid to mussaenosidic acid. *NsF3D3*, *NsF3H5*, and *NsF3H6* are more closely related to the *F3H* genes in plants such as *Echinacea purpurea* and *Glycine max* (**Supplementary Figure S9**).

UGD/UPD is the enzyme required to convert geniposidic acid to aucubin, whereas SQM is required to epoxidize aucubin further to generate catalpol. To determine whether the DEGs found in the transcriptome had *UGD* or *SQM* functions, we performed a phylogenetic analysis of known *UGD/UPD* and *SQM*. The results showed that four genes *NsUPD2*, *NsUPD3*, *NsUGD5*, and *NsUGD7* classified as *UGD/UPD*, *NsUPD3*, *NsUGD5*, and *NsUGD7*, respectively, are more closely related to the *UGD* gene in *Arabidopsis thaliana* (**Supplementary Figure S11**). Three genes *NsSQM1*, *NsSQM4*, and *NsSQM5* classified as *SQM*, *NsSQM1*, *NsSQM4*, and *NsSQM5*, respectively, are more closely related to the *TwSQM1* in *Tripterygium wilfordii* (**Supplementary Figure S12**). In summary, we speculate that these 11 genes may be functional genes responsible for the synthesis of P-I and P-II.

## 4 Discussion

*Neopicrorhiza scrophulariiflora* (Pennell) D.Y.Hong is a perennial medicinal plant with a long history of various pharmacological effects, including anti-inflammatory, anticancer, and antibacterial properties (Guo et al., 2020). Due to limited research on *N. scrophulariiflora*, the understanding of this plant is insufficient. To better protect and rationally utilize *N. scrophulariiflora* resources, it is necessary to conduct a combined analysis of its transcriptome and metabolome. Metabolomics is an effective tool for measuring metabolite composition in various plant tissues. Widely targeted metabolomics techniques have been used to identify and quantify metabolites in different plant species at different developmental stages and in other organs (Ren et al., 2022). The flavonoid component with the highest content in roots is isohyperoside, whereas the one with the highest content in leaves is 6-hydroxykaempferol-7-*O*-glucoside. Among the terpenoid compounds, P-II was the most abundant in roots, whereas P-I was the most abundant in leaves. Metabolomic analysis has identified several other iridoid glycosides besides P-I and P-II, including picrogentiosides B, picrosides IV, verminoside, specioside, and 6-feruloylcatalpol. In addition, several newly discovered compounds have been identified in *N. scrophulariiflora*, including amphicicide 8-*O*-feruloylharpagide, veronicoside, harpagoside, and rehmannioside B. However, the precursor compounds of P-I and P-II, aucubin and catalpol, may not have been detected due to their low content. It is worth noting that research on flavonoid compounds in *N. scrophulariiflora* is scarce, possibly because the underground parts (root tissues) are typically the focus of medicinal research, and there are almost no flavonoids in the roots. The aerial parts (leaf tissues), rich in flavonoids, have been studied less. Therefore, conducting metabolite analysis on the leaf tissues of *N. scrophulariiflora* is particularly important. According to the joint analysis of transcriptome and metabolome, in leaf and stem tissues, the accumulation of flavonoids increased, which may be due to the high expression of *NsCHS1*, *NsFLS1*, *NsFLS2*, *NsHCT4*, *NsHCT17*, *NsC3′H1*, and *NsC3′H3* genes in these tissues.

Iridoid glycosides are a class of compounds widely distributed in plants and can be further divided into iridoid alcohols, iridoid glycosides, and seco-iridoids based on their skeletal structures. P-I and P-II are the main pharmacologically active components of the iridoid glycosides found in *N. scrophulariiflora*. This study further explored the tissue-specific accumulation of P-I and P-II in *N. scrophulariiflora*. The results showed that iridoid glycosides exhibited different distribution patterns in various tissues of *N. scrophulariiflora*. P-I mainly accumulates in the leaf tissues, whereas P-II mainly accumulates in the root tissues. Furthermore, a comparative analysis of the content of iridoid glycosides in *N. kurroa* also confirmed that the content of P-I was higher in the leaves, whereas the content of P-II was higher in the roots (Pandit et al., 2013), which is consistent with the results of the present study. Based on this, we further analyzed the tissue specificity of the other compounds in *N. scrophulariiflora*. We found that flavonoids and phenolic acids primarily accumulated in the leaves and stems, whereas alkaloids, lignans, and coumarins mainly accumulated in the roots. Traditionally, the dried roots of *N. scrophulariiflora* have been used for medicinal purposes. However, the experimental results indicate that the leaves of *N. scrophulariiflora* also contain picrosides, with a particularly high concentration of P-I. This finding provides new perspectives and directions for studying the metabolic components of this plant. This not only helps to deepen the scientific understanding of *N. scrophulariiflora* but also promotes the conservation and sustainable utilization of its resources.

Although P-I and P-II have important medicinal values, their biosynthetic pathways in *N. scrophulariiflora* have not been clearly elucidated. The investigation of the terpenoid biosynthetic pathway in *N. kurroa* revealed that the expression levels of *2HFD*, *SQM*, and *DCH* genes were significantly higher in leaf tissue than in root tissue. In contrast, the expression levels of *F3D*, *UGD*, *UPD*, and *ACT* genes were significantly higher in root tissue than in leaf tissue. Our findings align with the gene expression patterns reported for the iridoid biosynthesis pathway in *N. kurroa*. In root tissues, a significant accumulation of P-II was observed, which is likely associated with the high expression of the *NsACT1*, *NsACT2*, *NsUGD7*, *NsUPD2*, *NsUPD3*, and *NsF3H6* genes. Conversely, in leaf tissues, a significant accumulation of P-I was detected, potentially attributed to the high expression of the *NsSQM4*, *NsSQM5*, and *Ns2HFD3* genes. Further functional studies in plants are necessary to comprehensively elucidate and confirm the biosynthetic pathways of these iridoid glycosides.

The results of the phylogenetic analysis ((**Supplementary Figure S9**-(**Supplementary Figure S12**)indicate that *NsF3D3*, *NsF3H5*, and *NsF3H6* are more closely related to the *F3H* genes in plants such as *Echinacea purpurea* and *Glycine max*. As a member of the cytochrome P450 superfamily in *Echinacea purpurea*, *F3H* catalyzes NADPH- and *O*2-dependent monooxygenase reactions. During anthocyanin biosynthesis, *F3H* specifically hydroxylates the 3′-position of the B-ring in naringenin and dihydrokaempferol (DHK) to yield eriodictyol and dihydroquercetin (DHQ), respectively. These intermediates play essential roles in the subsequent biosynthesis of anthocyanins and proanthocyanidins. The evolutionary tree analysis indicates that *NsF3D3*, *NsF3H5*, and *NsF3H6* catalyze the conversion of 8-epideoxyloganic acid to mussaenosidic acid in the biosynthetic pathway of *N. scrophulariiflora* iridoid glycosides (Gutierrez-Gonzalez et al., 2010; Wu, 2023); *Ns2HFD1* is more closely related to the *2HFD* gene in *Glycine max*. During isoflavone biosynthesis in *Glycine max*, *2HFD* catalyzes the dehydration of 2,5,7,4′-tetrahydroxyisoflavones or 2,7,4′-trihydroxyisoflavones, forming a double bond between C-2 and C-3 to yield genistein or daidzein, respectively. *Ns2HFD1* catalyzes the conversion of mussaenosidic acid to deoxygeniposidic acid in the biosynthetic pathway of *N. scrophulariiflora* iridoid glycosides (Akashi et al., 2005; Zhou et al., 2011); *NsUPD2*, *NsUPD3, NsUGD5*, and *NsUGD7* are more closely related to the *UGD* gene in *Arabidopsis thaliana*. In the biosynthesis pathway of *Arabidopsis* xylan, *UGD* irreversibly catalyzes the decarboxylation of UDP glucuronic acid (UDP GlcA) to form UDP Xyl. *NsUPD2*, *NsUPD3*, *NsUGD5*, and *NsUGD7* catalyze the conversion of geniposidic acid to bartsioside in the iridoid glycoside biosynthesis pathway of *N. scrophulariiflora* (Kuang et al., 2016). Similarly, *NsSQM1*, *NsSQM4*, and *NsSQM5* are involved in the biosynthetic pathway of iridoid glycosides in *N. scrophulariiflora*, specifically catalyzing the conversion of aucubin to catalpol (Liu et al., 2020). In summary, through phylogenetic tree analysis, the genes identified in this experiment are hypothesized to participate in the downstream pathway of iridoid glycoside biosynthesis in *N. scrophulariiflora*.

## 5 Conclusion

In this study, a widely targeted metabolomics approach was employed to identify 1,085 metabolites from three different tissues of *N. scrophulariiflora*, including 196 flavonoids and 63 terpenoids. The analysis revealed significant differences in flavonoid and iridoid glycoside content among the three tissues. To further understand the biosynthetic mechanisms underlying these differences, transcriptome data from these tissues were analyzed. A total of 61.63 Gb of clean data were obtained. The Q30 fraction of each sample was greater than or equal to 88.41%. By applying various bioinformatics methods such as differential expression gene (DEG) analysis, KEGG and GO enrichment analysis, clustering analysis, phylogenetic tree construction, and weighted gene co-expression network analysis (WGCNA), 43 candidate genes involved in P-I and P-II biosynthesis were successfully identified. Among these, 26 genes were significantly correlated with picroside content and their intermediate products. Furthermore, analysis of the flavonoid biosynthetic pathway revealed that genes related to flavonoid synthesis were highly expressed in the leaves and stems. Finally, 11 candidate genes were validated using quantitative real-time PCR (qRT-PCR), including *NsSQM1*, *NsSQM4*, *NsSQM5*, *NsUGD5*, *NsUGD7*, *NsUPD2*, *NsUPD3*, *Ns2HFD4*, *NsF3D3*, *NsF3H5*, and *NsF3H6*. We speculate that these genes may be involved in the biosynthetic pathway of iridoid glycosides, and their functions will be further validated in subsequent studies. The results of this study contribute to a comprehensive and systematic understanding of the composition and dynamic changes in iridoid compounds in *N. scrophulariiflora* and elucidate the molecular regulatory mechanisms of iridoid compound synthesis. The work lays the foundation for further development and utilization of the medicinal value of *N. scrophulariiflora*.

## Data Availability

The second-generation transcriptome data for this study are available from the NCBI Sequence Read Archive (SRA) database with the following accession number: PRJNA1171481.
