# Supplementary material for: Integrative analysis of metabolite and transcriptome reveals the biosynthetic pathway and candidate genes for iridoid glycoside biosynthesis in Neopicrorhiza scrophulariiflora (Pennell) D.Y.Hong
Source: Front Plant Sci. 2025 Feb 3;16:1527477. doi: 10.3389/fpls.2025.1527477 (PMC11830703; doi:10.3389/fpls.2025.1527477)
Supplement: Supplementary file 1 [file DataSheet1.docx]

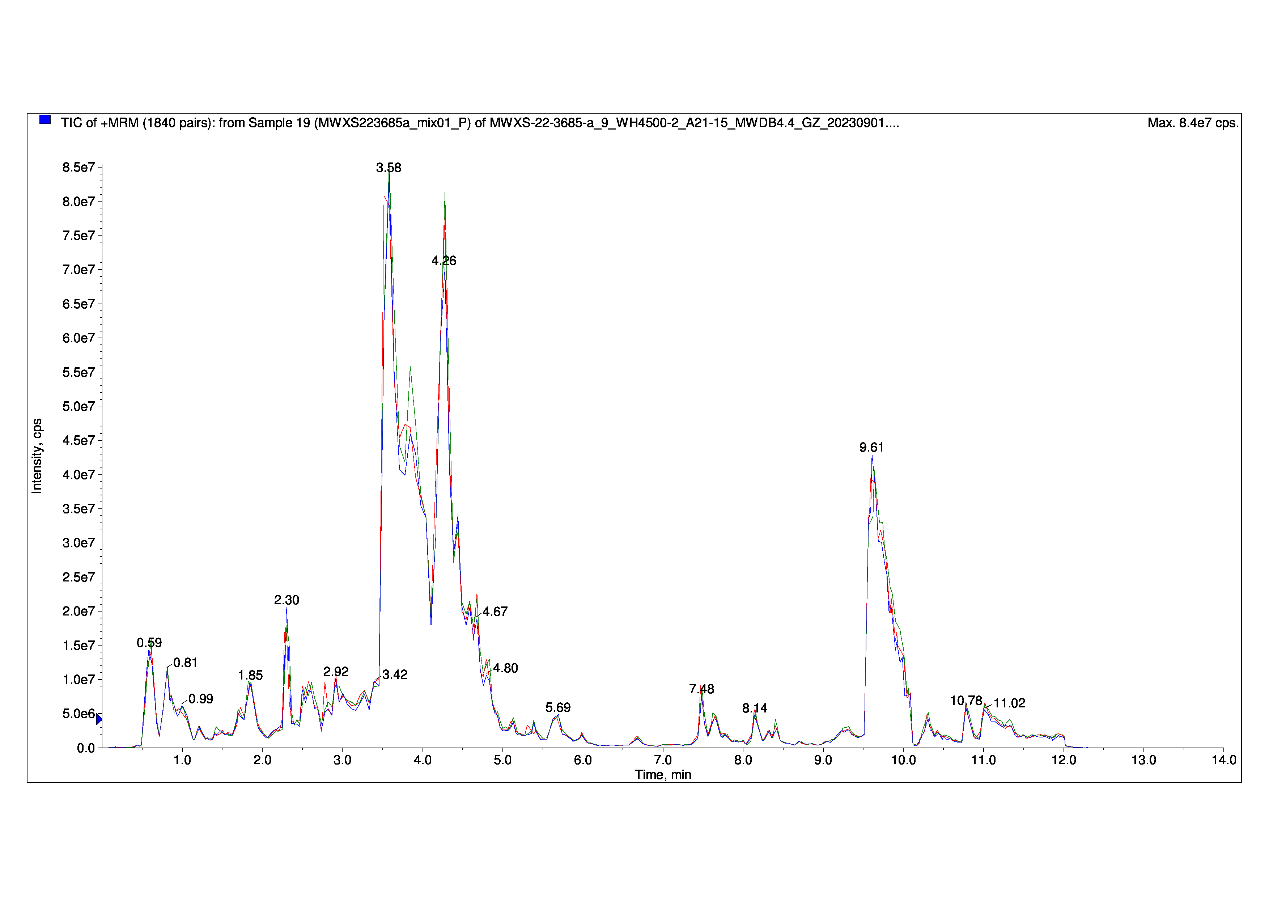

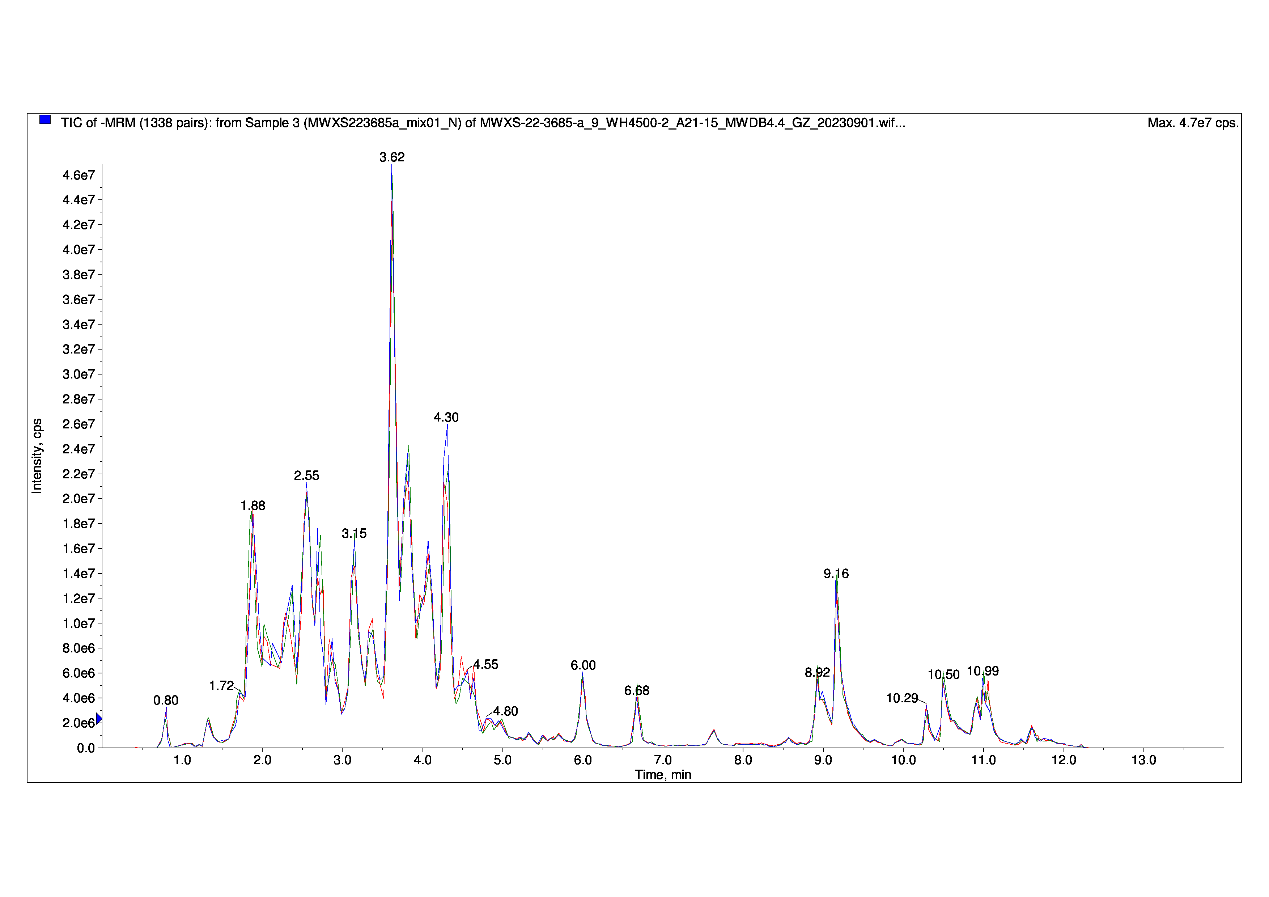
 Supplementary Fig. S1 Total Ion Current (TIC) of QC samples in negative and positive ion mode.


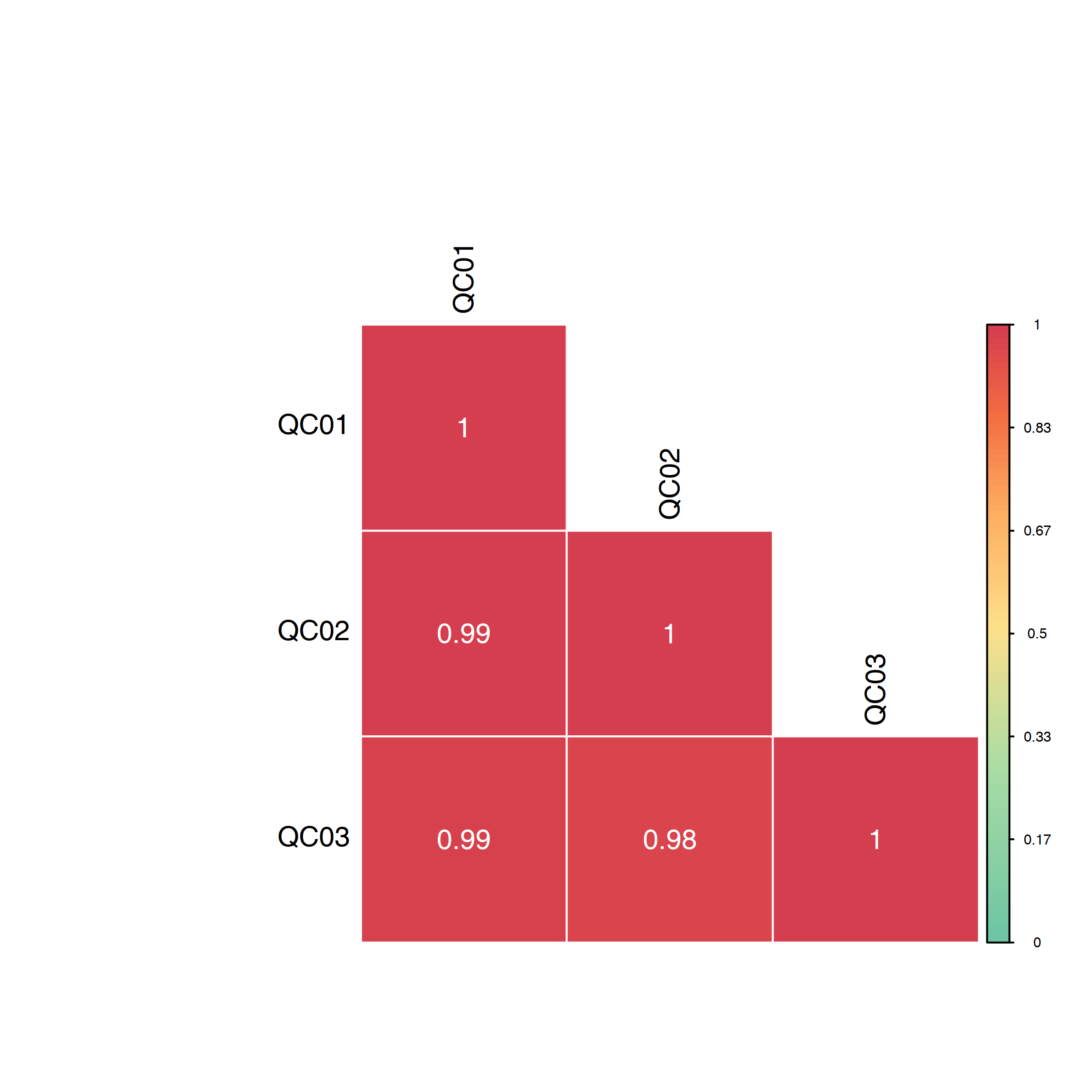


Supplementary Fig. S2 The correlation analysis using Pearson’s Correlation Coefficient (PCC).





Supplementary Fig. S3 Circular diagram of metabolite category composition.


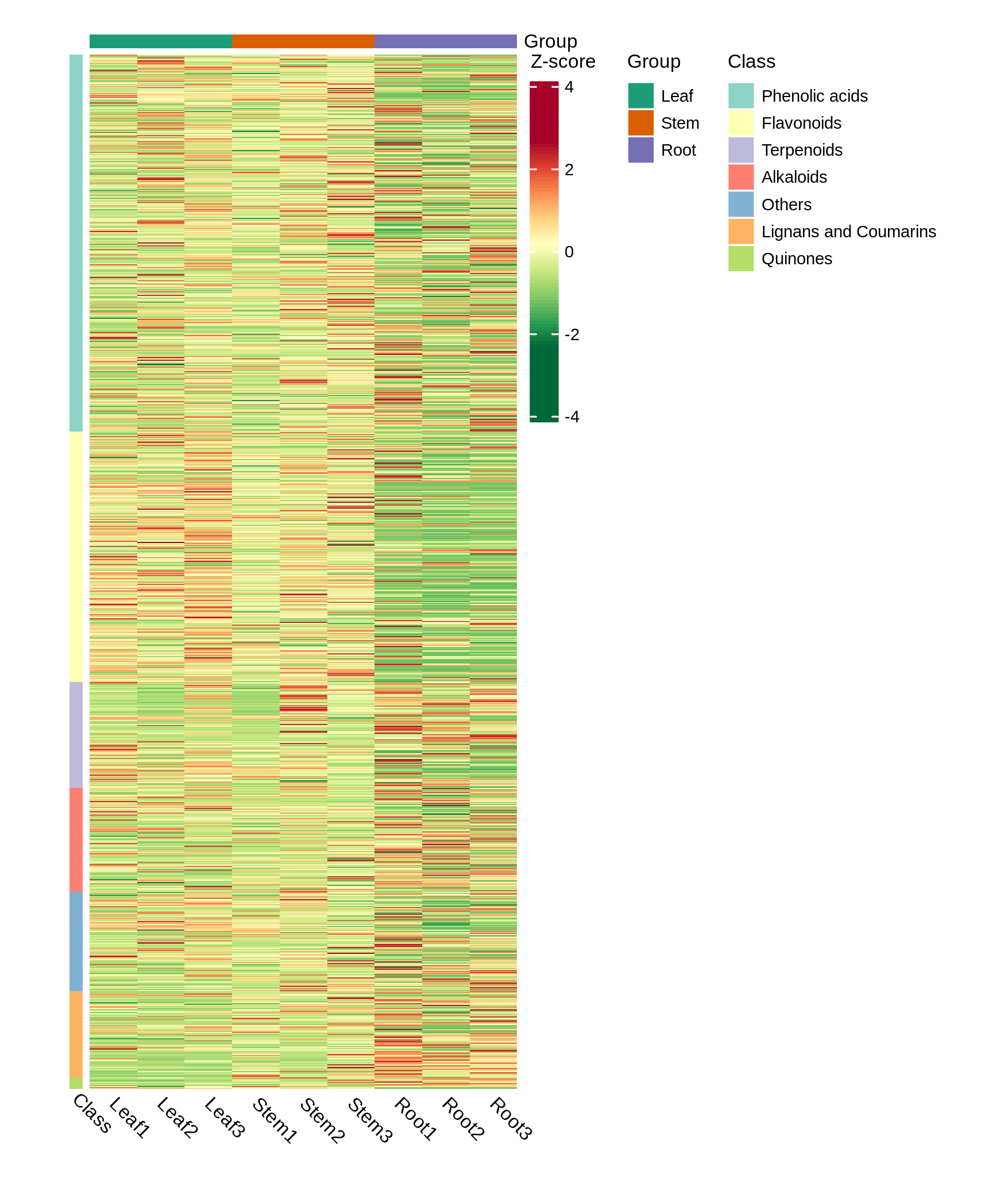


Supplementary Fig. S4 Heatmap based on hierarchical clustering analysis.


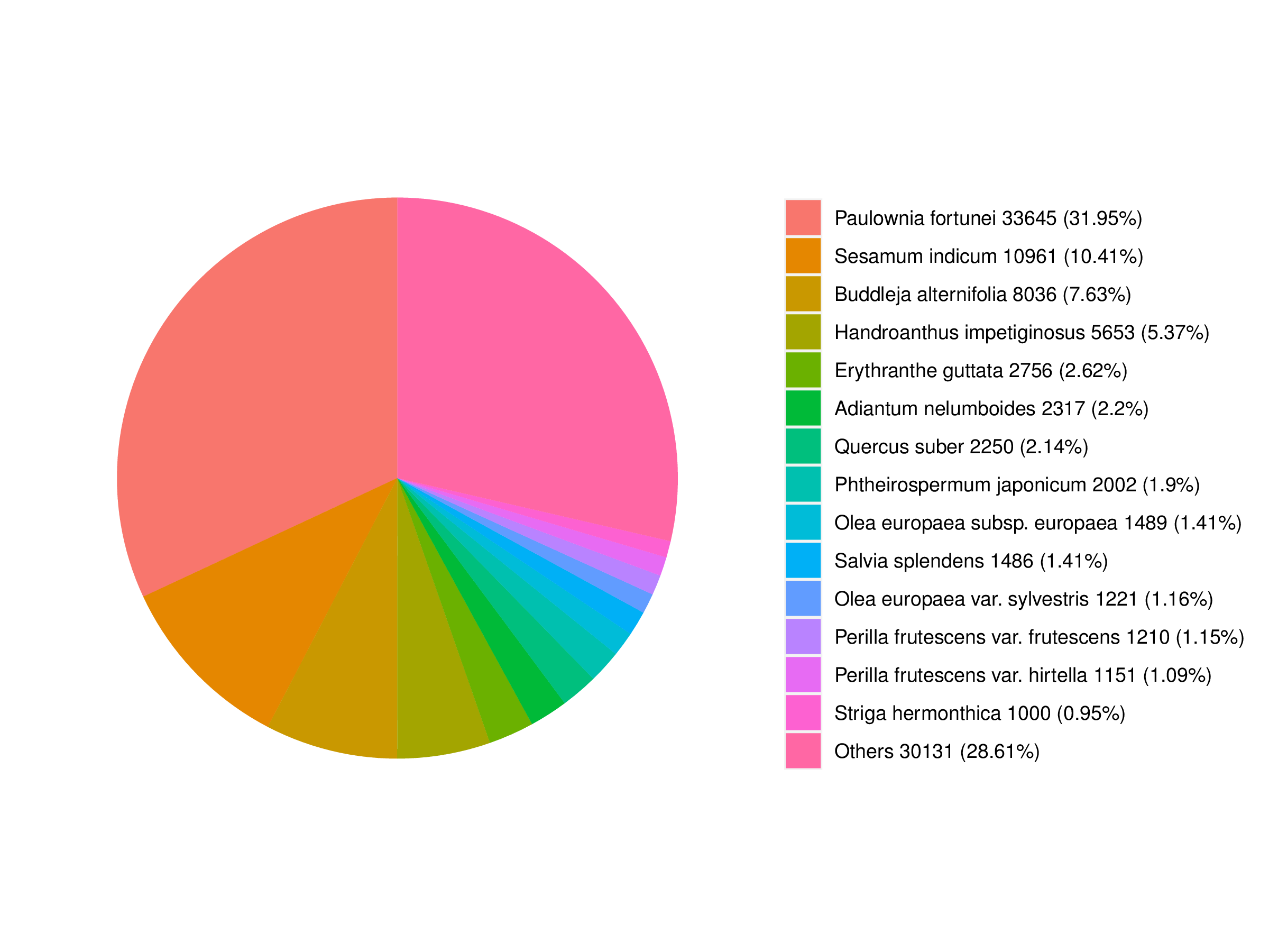
 Supplementary Fig. S5 The matched species of annotation of the genes of *Neopicrorhiza scrophulariiflora* (Pennell) to Nr database.


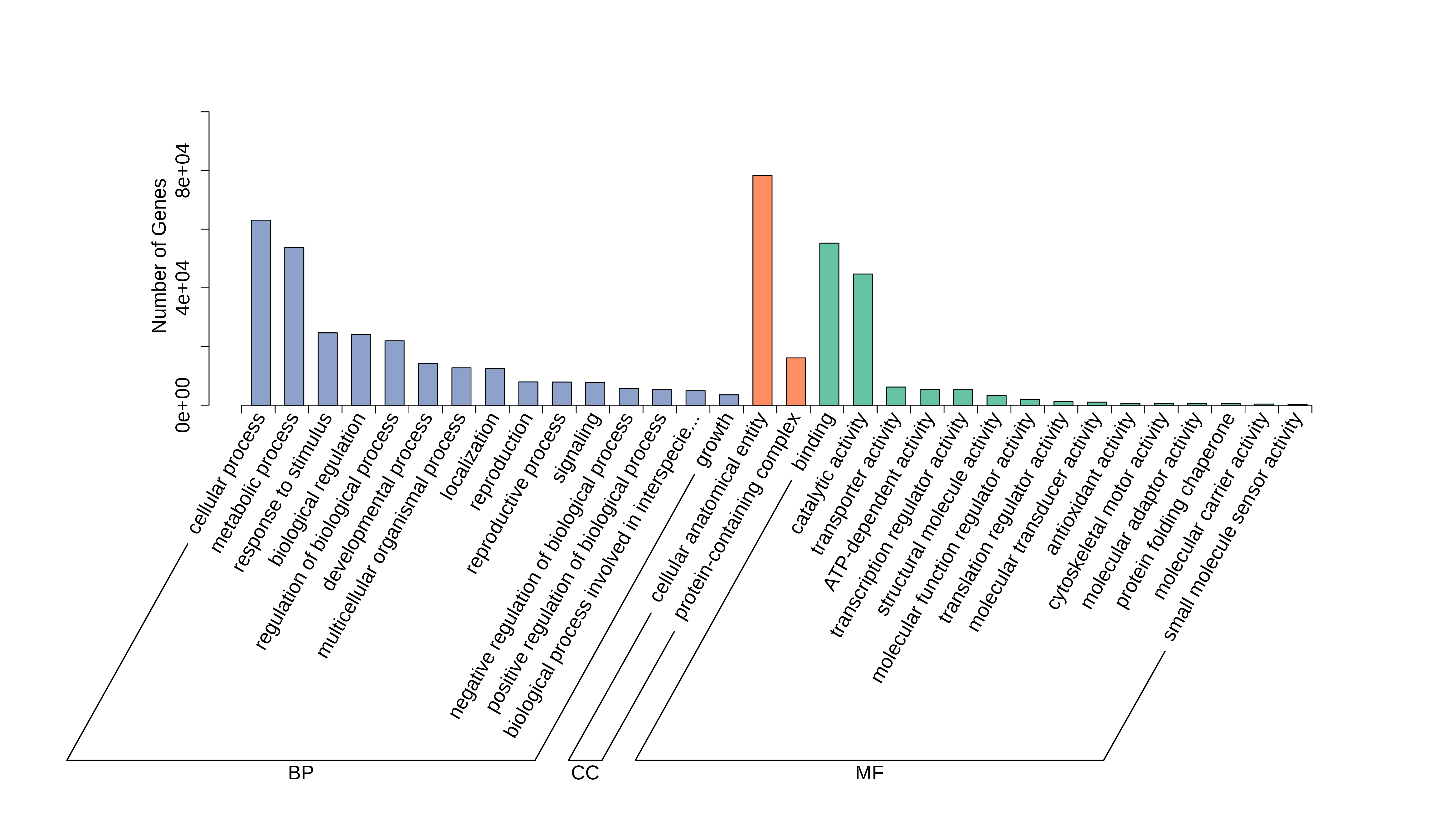


Supplementary Fig. S6 GO annotation of the genes of *Neopicrorhiza scrophulariiflora* (Pennell).


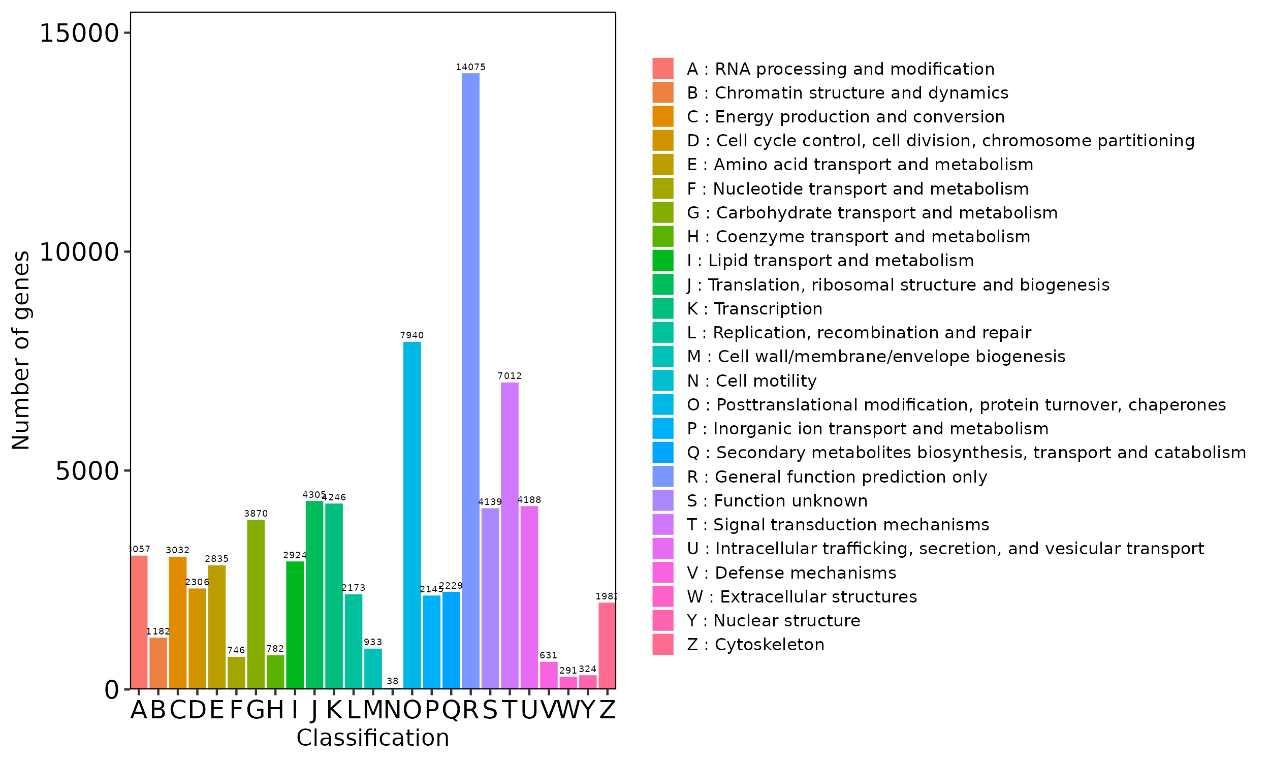


Supplementary Fig. S7 KOG annotation of the genes of *Neopicrorhiza scrophulariiflora* (Pennell).


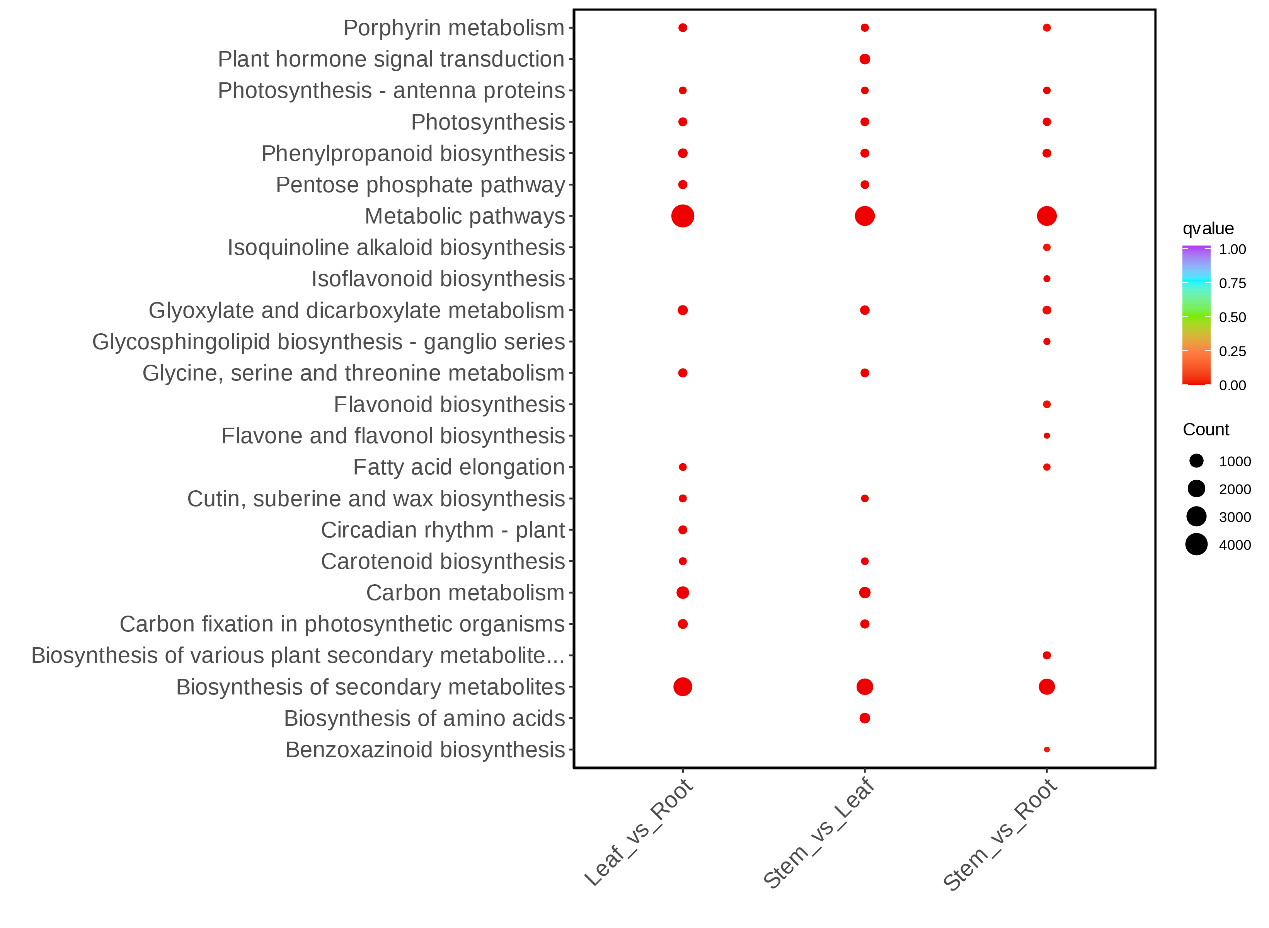
 Supplementary Fig. S8 KEGG enrichment analyses of the genes of *Neopicrorhiza scrophulariiflora* (Pennell).


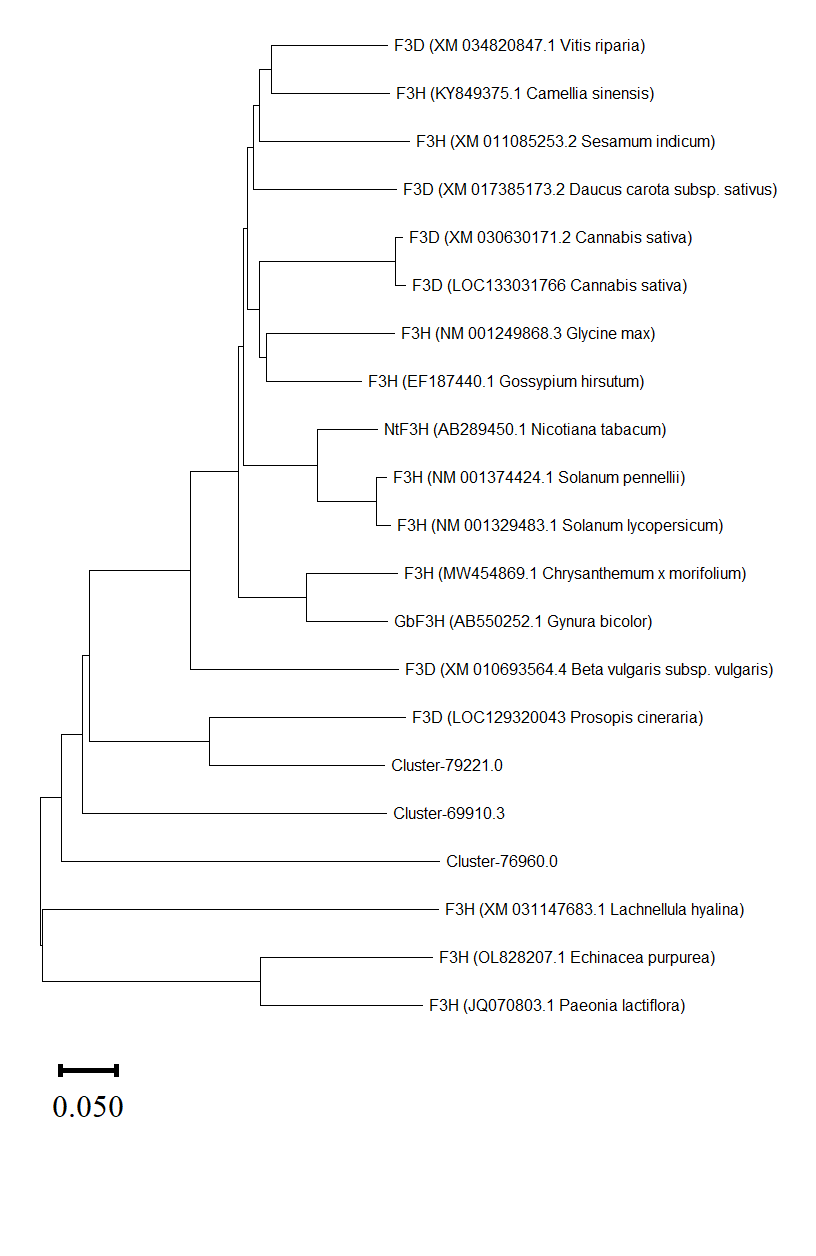
Supplementary Fig. S9 Phylogenetic analysis of F3D/F3H. The support values were obtained after 1,000 tests and indicated near the nodes.


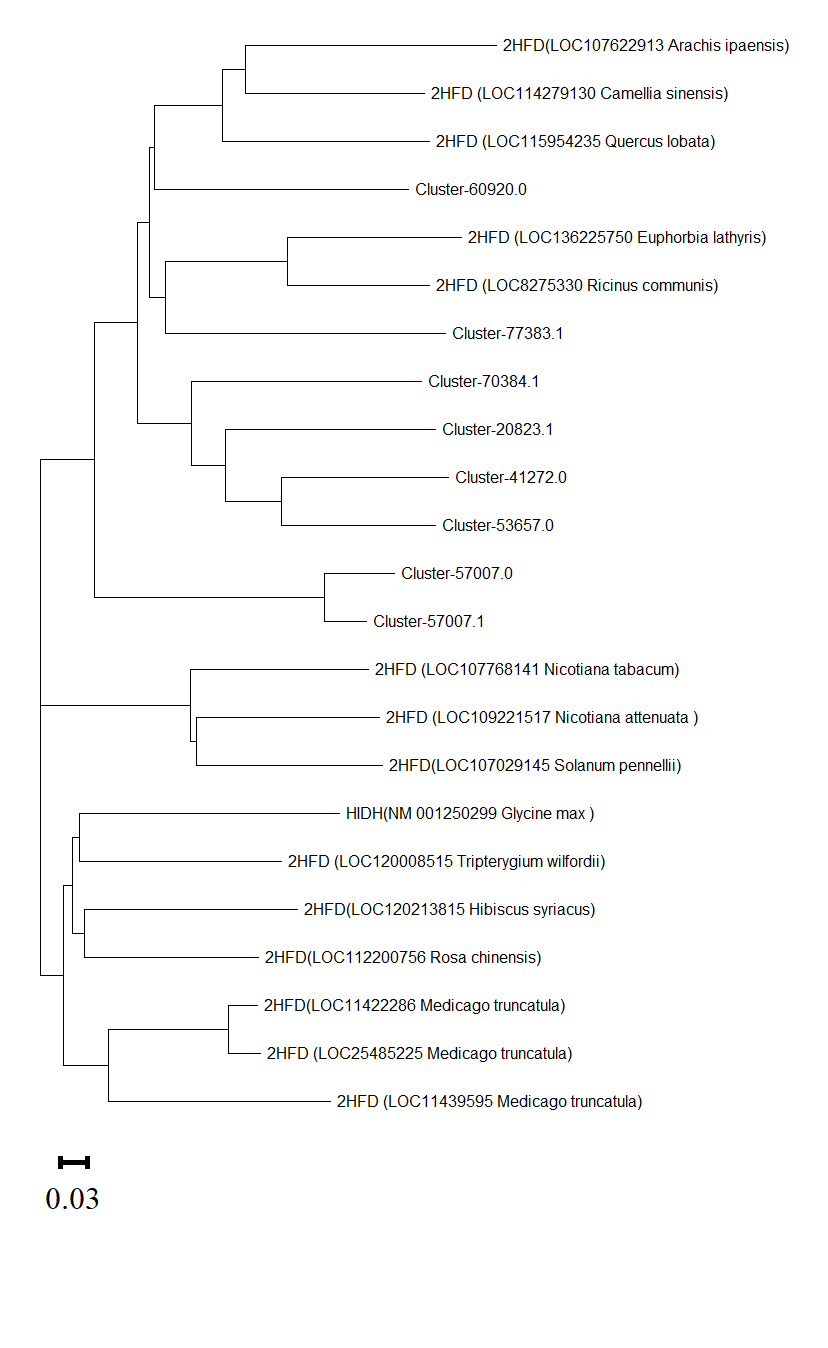


Supplementary Fig. S10 Phylogenetic analysis of 2HFD. The support values were obtained after 1,000 tests and indicated near the nodes.


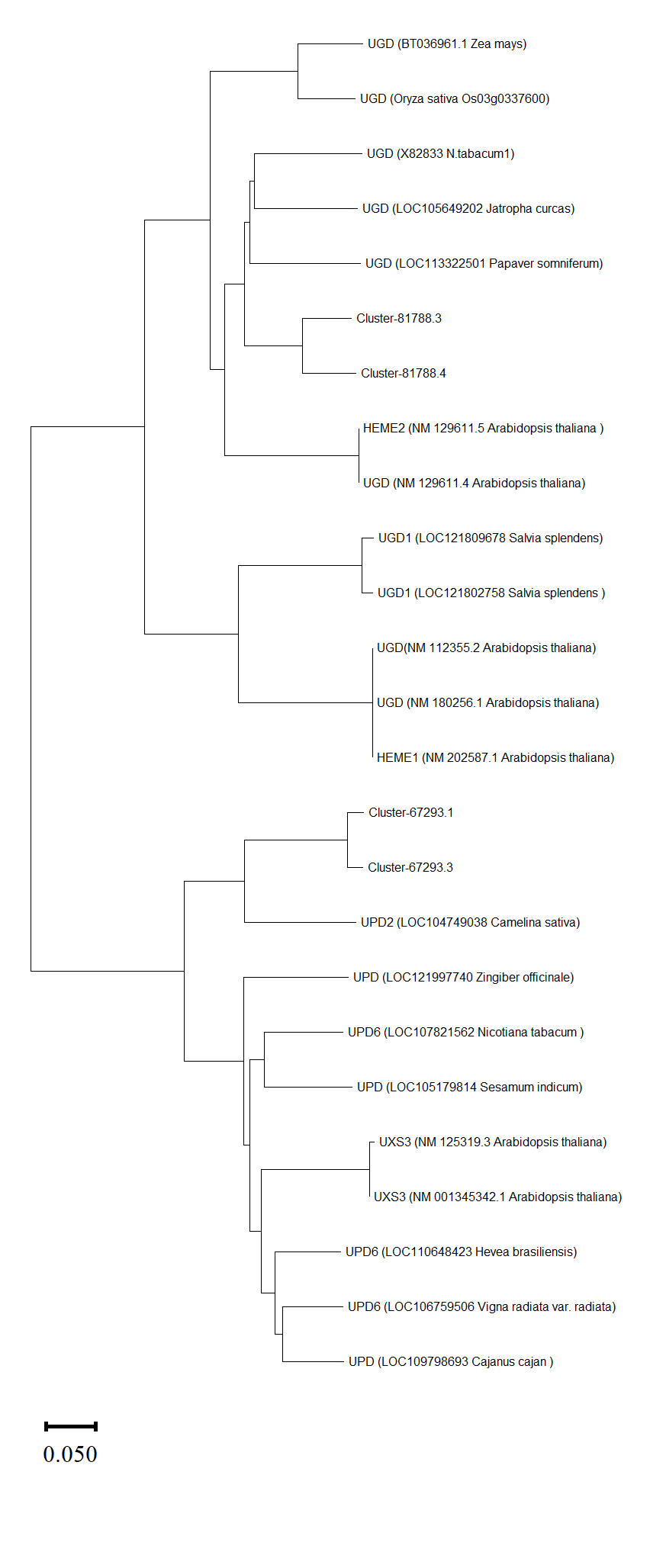


Supplementary Fig. S11 Phylogenetic analysis of UGD/UPD. The support values were obtained after 1,000 tests and indicated near the nodes.
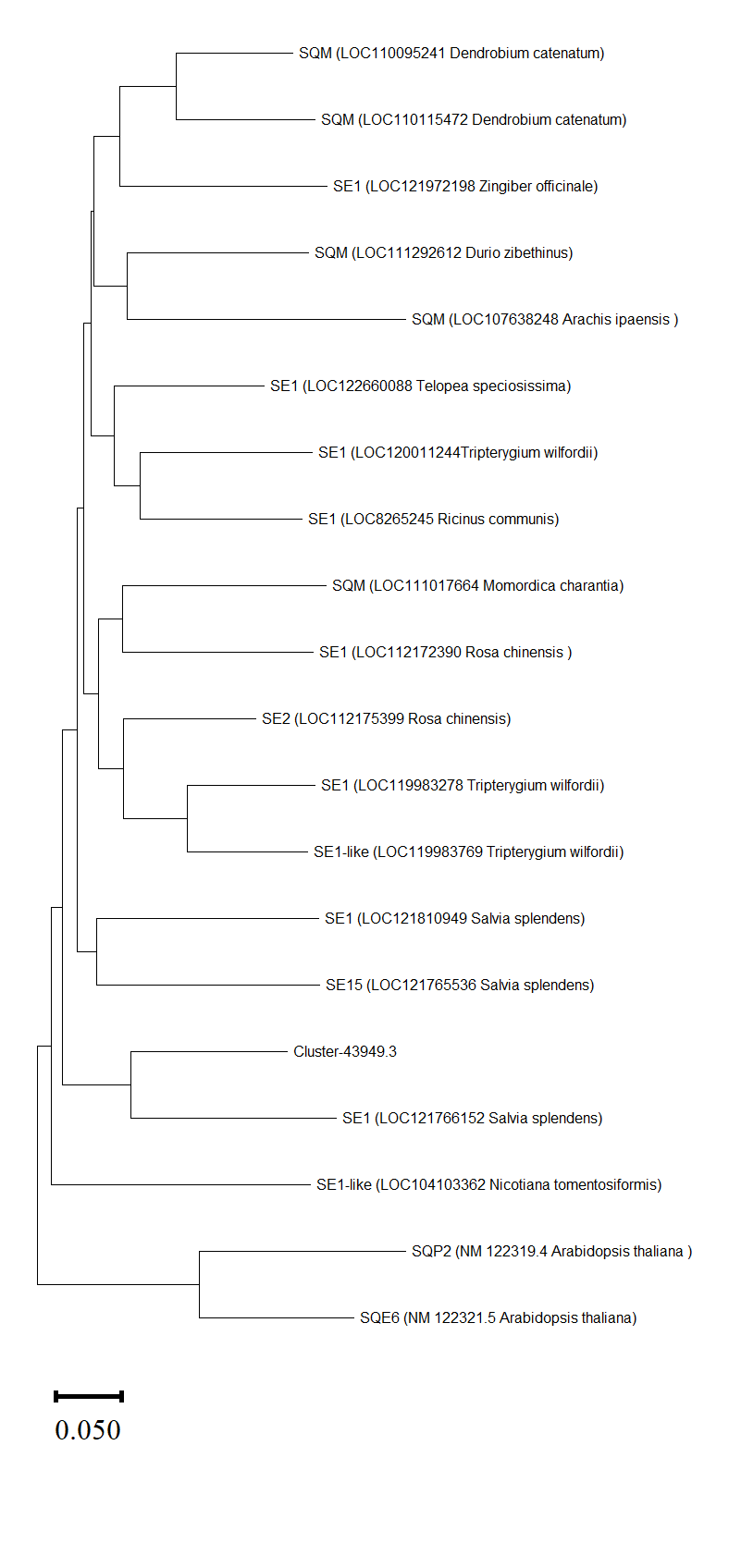


Supplementary Fig. S12 Phylogenetic analysis of SQM. The support values were obtained after 1,000 tests and indicated near the nodes.
